# Dual targeting of JAK2 and ERK interferes with the myeloproliferative neoplasm clone and enhances therapeutic efficacy

**DOI:** 10.1038/s41375-021-01391-2

**Published:** 2021-09-03

**Authors:** Sime Brkic, Simona Stivala, Alice Santopolo, Jakub Szybinski, Sarah Jungius, Jakob R. Passweg, Dimitrios Tsakiris, Stefan Dirnhofer, Gregor Hutter, Katharina Leonards, Heidi E. L. Lischer, Matthias S. Dettmer, Benjamin G. Neel, Ross L. Levine, Sara C. Meyer

**Affiliations:** 1 Department of Biomedicine, University Hospital Basel and University of Basel, Basel, Switzerland; 2 Division of Hematology, University Hospital Basel, Basel, Switzerland; 3 Department of Pathology, University Hospital Basel, Basel, Switzerland; 4 Interfaculty Bioinformatics Unit, University of Bern, Bern, Switzerland; 5 Swiss Institute of Bioinformatics, Lausanne, Switzerland; 6 Department of Pathology, University of Bern, Bern, Switzerland; 7 Laura and Isaac Perlmutter Cancer Center, New York University Langone, New York, NY USA; 8 Human Oncology and Pathogenesis Program and Leukemia service, Memorial Sloan Kettering Cancer Center, New York, NY USA

**Keywords:** Targeted therapies, Myeloproliferative disease

## Abstract

Myeloproliferative neoplasms (MPN) show dysregulated JAK2 signaling. JAK2 inhibitors provide clinical benefits, but compensatory activation of MAPK pathway signaling impedes efficacy. We hypothesized that dual targeting of JAK2 and ERK1/2 could enhance clone control and therapeutic efficacy. We employed genetic and pharmacologic targeting of ERK1/2 in *Jak2*V617F MPN mice, cells and patient clinical isolates. Competitive transplantations of *Jak2*V617F vs. wild-type bone marrow (BM) showed that ERK1/2 deficiency in hematopoiesis mitigated MPN features and reduced the *Jak2*V617F clone in blood and hematopoietic progenitor compartments. ERK1/2 ablation combined with JAK2 inhibition suppressed MAPK transcriptional programs, normalized cytoses and promoted clone control suggesting dual JAK2/ERK1/2 targeting as enhanced corrective approach. Combined pharmacologic JAK2/ERK1/2 inhibition with ruxolitinib and ERK inhibitors reduced proliferation of *Jak2*V617F cells and corrected erythrocytosis and splenomegaly of *Jak2*V617F MPN mice. Longer-term treatment was able to induce clone reductions. BM fibrosis was significantly decreased in *MPL*W515L-driven MPN to an extent not seen with JAK2 inhibitor monotherapy. Colony formation from *JAK2*V617F patients’ CD34^+^ blood and BM was dose-dependently inhibited by combined JAK2/ERK1/2 inhibition in PV, ET, and MF subsets. Overall, we observed that dual targeting of JAK2 and ERK1/2 was able to enhance therapeutic efficacy suggesting a novel treatment approach for MPN.

## Introduction

Myeloproliferative neoplasms (MPN) are clonal hematopoietic stem cell disorders characterized by excessive output of mature myeloid cells and inherent risk for leukemic transformation [1]. MPN subtypes show distinct clinical phenotypes; polycythemia vera (PV) primarily characterized by erythrocytosis, essential thrombocythemia (ET) featuring thrombocytosis, and myelofibrosis (MF) typified by progressive bone marrow (BM) fibrosis inducing cytopenias [2]. These MPN subsets share dysregulated JAK2 signaling [3] constitutively activated by somatic mutations in JAK2, thrombopoietin receptor MPL or the chaperone calreticulin (CALR) [4]. JAK2 inhibitors represent a standard treatment in MPN approved for MF and PV [5]. Ruxolitinib, fedratinib or JAK2 inhibitors in development provide benefits including reduction of splenomegaly and symptoms. However, disease-modifying activity of clinical JAK2 inhibitors has remained modest [6].

MAPK pathway signaling including the sequential RAF, MEK, and ERK kinases, is involved in many cancers including hematologic malignancies, and has been suggested for targeting in several disease settings [7–12]. In MPN, inhibition of BRAF or MEK has been explored in combination with JAK2 inhibitors in vitro [13–15] and RAS/MAPK pathway gene mutations were shown to impact on response to treatment and outcome of MPN patients [16, 17]. It has been shown that MAPK pathway signaling functionally interferes with JAK2 inhibition in MPN, thus limiting the corrective potential of JAK2 inhibitors [18–20]. While we have reported that MAPK signaling remains activated upon ruxolitinib therapy via PDGFRα signaling [18], in-depth analyses of global JAK2 signaling have elucidated cell-intrinsic mechanisms of MAPK pathway activation via splicing factor YBX1 [19, 20]. An important role of MAPK pathway activation as a therapeutic target in MPN has been confirmed by the efficacy of combined JAK2 and MEK inhibition in preclinical models and patient cells observed with clinical and preclinical compounds including binimetinib, selumetinib, trametinib, and PD0325901 [18, 19]. These findings highlight that MAPK pathway signaling must be addressed to enhance therapeutic efficacy of JAK2 inhibition in MPN.

Knowledge is scarce about the potential of targeting alternative MAPK pathway components other than BRAF or MEK, which might represent more optimal targets to control MPN. ERK1/2 are distal to MEK1/2 in the MAPK pathway, which could render ERK inhibition less prone for escape from therapeutic inhibition by adaptive signaling changes [7]. ERK1/2 directly act on multiple immediate downstream targets implicated in proliferation and survival, giving them a “switch” function for concerted MAPK pathway-driven effector programs. Importantly, ERK1/2 have been shown to mediate essential functions for hematopoiesis with ERK1/2 ablation inducing cytopenias and decreasing hematopoietic progenitor clones in normal hematopoiesis [21, 22]. We hypothesized that targeting ERK could be advantageous to enhance clone control and increase therapeutic efficacy of JAK2 inhibition in MPN. Thus, we evaluated dual targeting of JAK2 and ERK1/2 as corrective approach in MPN preclinical models, cells and patient clinical isolates using genetic and pharmacologic approaches.

## Materials and methods

### Mouse models

Genetic and pharmacologic targeting of ERK1/2 was performed in *Jak2*V617F knock-in mice [23]. For genetic studies, *Jak2*V617F Mx-1-Cre C57BL/6 mice [23] were crossed with *Erk1*^−/−^*Erk2*^fl/fl^ C57BL/6 [21]. *Jak2*V617F *Erk1*^−/−^*Erk2*^fl/fl^ Mx-1-Cre CD45.2 mice aged 6–8 weeks were induced by poly I:C (pIpC) [21], BM was mixed 1:1 with BM from *Jak2* wild-type (WT) CD45.1 mice aged 8–10 weeks and a total of 2 × 10^6^ cells transplanted into lethally irradiated female CD45.1 C57BL/6 aged 8–10 weeks (*n* = 10–12/group). For secondary transplantation, 2 × 10^6^ pooled BM cells from 4–5 primary recipients were injected into lethally irradiated CD45.1 secondary recipients (*n* = 10–12/group). For inhibitor studies, CD45.1 C57BL/6 female recipient mice competitively transplanted with *Jak2*V617F Vav-Cre CD45.2 BM [23–25] mixed 1:1 with *Jak2*WT CD45.1 BM (total 4 × 10^6^ cells) were randomized 16–18 weeks post-transplant according to blood counts and treated by gavage for 1–4 weeks (*n* = 6–8/group). *Jak2*V617F Vav-Cre CD45.2 donors were 11–33 weeks old. For evaluation of fibrosis, a *MPL*W515L model was used. CD117-enriched (Miltenyi) BM from 8–10 week old Balb/c mice was transduced with retroviral supernatant containing MSCV-*hMPL*W515L-IRES-GFP and 600.000 GFP^+^ cells transplanted into lethally irradiated Balb/c [25, 26]. Randomization was 4 weeks post-transplant according to blood counts and treatment for 1–4 weeks (*n* = 3–6/group). For studies of combined genetic targeting of ERK1/2 and inhibition of JAK2, CD45.1 C57BL/6 female recipients of *Jak2*V617F *Erk1*^−/−^*Erk2*^fl/fl^ Mx-1-Cre CD45.2 BM mixed 1:1 with *Jak2*WT CD45.1 BM were treated with pIpC 5 weeks after transplantation as described [21] and ruxolitinib for 2 weeks (*n* = 4–5/group). Group size was estimated from previous data. In vivo experiments were not blinded and were all confirmed in a second experiment on a separate mouse cohort. Histopathology analyses and fibrosis grading were performed by a blinded hematopathologist [18]. Images were taken on Olympus BX43 (cellSense 1.6). Animal care, all animal procedures and experiments were in strict adherence to Swiss laws for animal welfare and approved by Swiss Cantonal Veterinary Office of Basel-Stadt.

### Inhibitors

The ERK1/2 inhibitor LTT462 and ruxolitinib were provided by Novartis via Material Transfer Agreement, MK-8353, SCH772984 and DEL-22379 purchased from Sellekchem. LTT462 was administered by gavage at 75 mg/kg qd, ruxolitinib at 60 mg/kg bid and MK-8353 at 30 or 40 mg/kg bid. Inhibitors for in vitro use were stored at −20 °C in DMSO.

### Flow cytometry

Myelo-erythroid progenitors were assessed using lineage markers, Sca-1, c-Kit, CD41, CD150, CD48, CD16/32, CD105, CD71 and Ter119 on LSRFortessa(BD). Allele burden was determined as fraction of CD45.2 cells in *Jak2*V617F or percentage of GFP^+^ cells in *MPL*W515L models.

### Colony formation

Colonies were scored 10d after plating mouse BM or spleen cells into MethoCult (STEMCELL,#03434) including erythroid (BFU-E), granulocytic-macrophage (CFU-GM) and granulocyte-erythroid-macrophage-megakaryocyte (CFU-GEMM) subtypes.

### RNA expression

RNA was extracted with Nucleospin RNA-Plus (Macherey-Nagel) and reverse-transcribed with high-capacity cDNA reverse transcription (Applied Biosystems). Expression of ERK1/2 targets and cytokines was determined by Nanostring according to manufacturer’s protocol and analyzed using NanoStringDiff software [27]. Validation by qRT-PCR was in triplicates on ABI7500 thermocycler using SyBR Green (Applied Biosystems). Primers are listed in Supplementary Table 1.

### Cell lines and proliferation assays

Ba/F3 cells (ATCC) stably expressing *Jak2*V617F along with erythropoietin receptor (EPOR) were cultured in RPMI1640/10%FCS, supplemented with 10 U/ml EPO if expressing *Jak2*WT. ERK1/2-deficient *Jak2*V617F cells were generated by transducing ERK1/2-specific shRNA in pLKO-Tet-On vector, puromycin selection and doxycycline induction (Supplementary Table 2). Proliferation was assessed upon ERK1/2 knockdown and/or exposure to inhibitors for 48 h using Cell viability luminescent assay (Promega). Experiments were repeated independently three times. IC_50_ was determined with Prism 9.0.

### Signaling analyses

Cells were exposed to inhibitor for 4 or 24 h and lysed in presence of Protease Arrest (EMD) and Phosphatase Inhibitor (Calbiochem). Total protein was normalized by BCA quantitation, separated on 4–12% Bis-Tris gels (Invitrogen) and blots probed for phospho-/total-ERK1/2, phospho-/total-MEK1/2, phospho-/total-RSK3, DUSP6, c-MYC, phospho-/total-STAT5, phospho-/total-STAT3 and/or actin. For analysis after in vivo treatment, mice were sacrificed 2 h after gavage. Splenocytes were lysed followed by electrophoresis and immunoblotting as described above.

### Patient samples

Collection of blood, BM samples, and clinical data from MPN patients was approved by the Ethik-Kommission Beider Basel. Written informed consent was obtained from all subjects in accordance with the Declaration of Helsinki. Diagnosis of MPN was according to the revised WHO criteria. For colony formation assays, CD34^+^ PBMCs or BM cells were plated at 3.000 or 150.000 cells/well, respectively, into MethoCult (STEMCELL, #04435/#04034) with 0.25 μM ruxolitinib and/or 0.25–2.5 μM LTT462. Colony number and subtypes including erythroid (CFU-E, BFU-E), granulocyte-macrophage (CFU-GM) and granulocyte-erythroid-macrophage-megakaryocyte (CFU-GEMM) were scored after 10d. For signaling analyses, PBMCs were serum-starved in αMEM/1% BSA and exposed to 0.25 μM ruxolitinib and/or 2.5 μM LTT462 for 16 h. Cells were lysed followed by electrophoresis and immunoblotting as described above.

### Statistics

Results are shown as mean ± SEM or SD. Statistical significance was assessed by one-way ANOVA with Bonferroni post-hoc multiple comparison testing or two-tailed unpaired Student *t* test (Prism 9.0). *P* values ≤ 0.05 were considered significant. No samples/animals were excluded from analysis.

## Results

### Genetic targeting of ERK1/2 mitigates the MPN phenotype and impairs the fitness of the Jak2V617F clone

To assess the role of ERK1/2 for hematopoiesis in *Jak2*V617F MPN settings, we crossed conditional *Jak2*V617F knock-in [23] to *Erk1*^−/−^*Erk2*^fl/fl^ mice [21] expressing Mx-1-Cre recombinase. Poly I:C induction of *Jak2*V617F *Erk1*^−/−^ *Erk2*^fl/fl^ Mx-1-Cre^+^ mice resulted in ERK1/2 deficiency of hematopoietic cells as described [21] (Supplementary Fig. 1A). ERK1/2 deficiency moderated the erythrocytosis of *Jak2*V617F mice and mitigated additional MPN features, specifically leukocytosis, splenomegaly and BM fibrosis. Hematopoietic progenitor cells including Lin^−^Sca1^+^Kit^+^ (LSK) and Lin^−^Sca1^−^Kit^+^ multipotent myeloid progenitors (MP) were reduced with impaired myeloid colony formation from BM and spleen cells ex vivo. Other organs were unaffected consistent with *Jak2*V617F expression and ERK1/2 deficiency in hematopoiesis (Supplementary Fig. 1B-R).

**Fig. 1:**
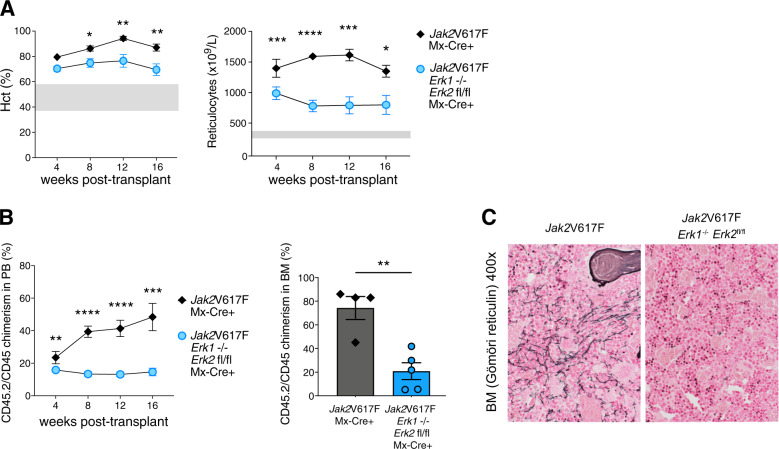
Genetic targeting of ERK1/2 mitigates the MPN phenotype and impairs the *Jak2*V617F clone. *Jak2*V617F CD45.2 bone marrow (BM) with intact (gray) or deficient (blue) ERK1/2 was transplanted in 1:1 ratio with *Jak2*WT CD45.1 BM into CD45.1 C57BL/6 recipient mice (for schema see Supplementary Fig. 3A). **A** Erythrocytosis reflected by increased hematocrit and reticulocytes was moderated by ERK1/2 deficiency in *Jak2*V617F settings (*n* = 10–11/group, shaded areas represent normal range). **B** CD45.2/CD45 chimerism reflecting *Jak2*V617F allele burden was significantly reduced by ERK1/2 deficiency in peripheral blood (*n* = 10–11/group) and BM 16 weeks after transplantation (*n* = 4–5/group). **C** BM fibrosis assessed by reticulin (Gömöri) staining 30 weeks after transplantation was not evident in ERK1/2 deficient *Jak2*V617F mice, whereas fibrosis was detected in *Jak2*V617F mice with intact ERK1/2 as reflected by fibrosis grading by a specialized hematopathologist blinded for the respective genotypes (*n* = 3/group, for grading see Supplementary Fig. 3). Original magnification ×400. Results from one of two independent experiments are shown. Data are displayed as mean ± SEM and analyzed by two-tailed Student *t* test. *P ≤ 0.05, **P ≤ 0.01, ***P ≤ 0.001, ****P ≤ 0.0001.

As ERK1/2 deficiency affected MPN hematopoiesis, we sought to establish a model of a *Jak2*V617F MPN clone along with normal *Jak2*WT hematopoietic cells similar to the situation in patients. As ERK1/2 deficiency did not impede engraftment of *Jak2*V617F BM (Supplementary Fig. 2), we employed competitive transplantations of *Jak2*V617F CD45.2 BM with intact or deficient ERK1/2 alongside *Jak2*WT CD45.1 BM in 1:1 ratio (Supplementary Fig. 3A). In this setting, ERK1/2 deficiency significantly moderated erythrocytosis as reflected by reduced hematocrit (Hct), red blood cells and reticulocytes without inducing cytopenia (Fig. 1A, Supplementary Fig. 3B-D), and substantially decreased MPN clones (Fig. 1B). *Jak2*V617F allele burden reflected by the fraction of CD45.2 cells was significantly reduced in blood and BM of mice transplanted with ERK1/2-deficient *Jak2*V617F cells compared to mice receiving cells with intact ERK1/2 and this effect was seen in myeloid, erythroid and megakaryocytic progenitor compartments (Supplementary Fig. 3E-F). BM cellularity and hematopoietic stem/progenitor cell frequencies were not compromised (Supplementary Fig. 3G-I). Hematopoietic stem/progenitor cell function was maintained as shown by similar number of colonies from mice with deficient or intact ERK1/2; however, the contribution from the *Jak2*V617F clone to colony formation was significantly reduced upon ERK1/2 deficiency (Supplementary Fig. 3J-K). Analogously, similar number of spleen-derived colonies were observed, with reduced *Jak2*V617F mutant cell contribution in ERK-deficient settings (Supplementary Fig. 3L-M). Of note, ERK1/2 deficiency prevented development of BM fibrosis (Fig. 1C, Supplementary Fig. 3N). Long-term follow-up to 30 weeks confirmed that mitigation of the MPN phenotype and decrease of the MPN clone were maintained in ERK1/2-deficient settings (Supplementary Fig. 3O-U). Corrective effects of ERK1/2 deficiency on the MPN phenotype were enhanced in secondary recipients with normalization of red cell parameters (Supplementary Fig. 4A-E) and progressive loss of the *Jak2*V617F clone in blood and BM stem/progenitor populations (Supplementary Fig. 4F-J). Colony formation from BM and spleen was mostly from *Jak2*WT progenitor cells in ERK1/2 deficient settings, while 60–70% of colonies were *Jak2*V617F positive in mice with intact ERK1/2 (Supplementary Fig. 4K-N). Emergence of fibrosis was prevented and BM cellularity maintained with normalization of splenomegaly while corrective effects persisted at longer term (Supplementary Fig. 4O-V).

### Genetic targeting of ERK1/2 enhances corrective effects of JAK2 inhibition with ruxolitinib in Jak2V617F mice

**Fig. 2:**
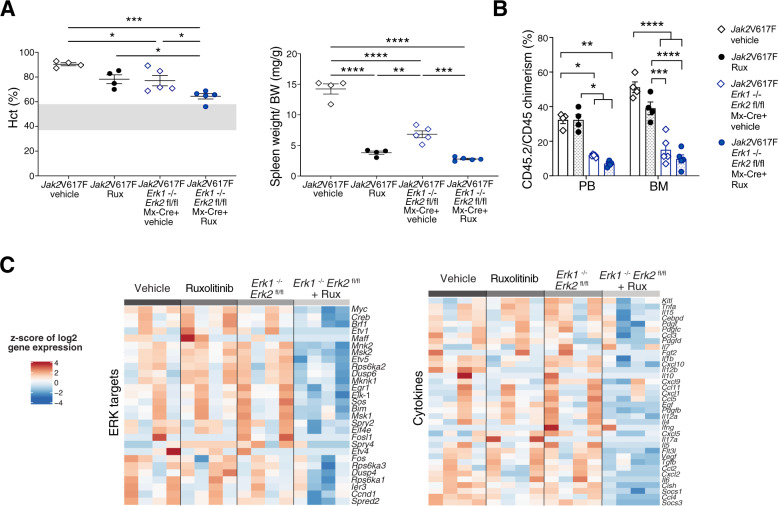
Genetic targeting of ERK1/2 enhances corrective effects of JAK2 inhibition with ruxolitinib in *Jak2*V617F mice. **A** Erythrocytosis reflected by increased hematocrit was moderated by ERK1/2 deficiency or ruxolitinib and ERK1/2 deficient settings enhanced ruxolitinib effects as indicated by near-normalized values upon combined treatment (*n* = 4-5/group, left panel). Ruxolitinib corrected splenomegaly without additional benefit by targeting ERK1/2 (right panel). **B** CD45.2/CD45 chimerism reflecting *Jak2*V617F allele burden was significantly reduced by targeting ERK1/2 in peripheral blood and BM and reductions were most profound with combined ERK1/2 deficiency and ruxolitinib (*n* = 4–5/group). **C** Expression of 28 ERK1/2 downstream targets (left panel) as well as of 36 cytokines (right panel) in BM as assessed by Nanostring analysis was most effectively reduced by combined targeting of ERK1/2 and ruxolitinib (*n* = 4/group). Results from one of two independent experiments are shown. Data are shown as mean ± SEM and analyzed by one-way ANOVA. *P ≤ 0.05, **P ≤ 0.01, ***P ≤ 0.001, ****P ≤ 0.0001.

Since genetic targeting of ERK1/2 was able to interfere with MPN phenotype and clone size, we hypothesized that ERK1/2 deficiency in MPN cells could enhance therapeutic effects of JAK2 inhibition by ruxolitinib. To explore additional benefits of dual JAK2 and ERK1/2 targeting, we combined five daily doses of pIpC to induce ERK1/2 deficiency with 60 mg/kg ruxolitinib bid or vehicle for 2 weeks in mice competitively transplanted with *Jak2*V617F *Erk1*^−/−^*Erk2*^fl/fl^ Mx-1-Cre^+^ and *Jak2*WT BM (Supplementary Fig. 5A). Ruxolitinib reduced erythrocytosis and normalized splenomegaly as expected (Fig. 2A, Supplementary Fig. 5B) along with modest effects on MPN clone size (Fig. 2B). However, dual targeting of JAK2 and ERK1/2 via genetically induced ERK1/2 deficiency enhanced reduction of erythrocytosis in *Jak2*V617F settings, while no cytopenia occurred (Supplementary Fig. 5C-D). Genetic targeting of ERK1/2 was able to induce significant reductions of *Jak2*V617F clones in presence and absence of ruxolitinib therapy and most pronounced effects were seen when both JAK2 and ERK1/2 were targeted (Fig. 2B). These effects observed in BM and in myeloid and erythroid progenitor subsets were similarly seen in the spleen (Supplementary Fig. 5E-J). With respect to ERK1/2 downstream effectors, dual targeting of both JAK2 and ERK1/2 consistently decreased expression of 28 ERK1/2 target genes. Expression levels of inflammatory cytokines, which were moderated by ruxolitinib, were more clearly reduced upon dual JAK2 and ERK1/2 targeting (Fig. 2C). Overall, double targeting showed the most pronounced effects on ERK1/2 target and inflammatory cytokine expression profiles evidenced by principal component analysis (Supplementary Fig. 5K). These findings credential a combinatorial benefit of dual JAK2 and ERK1/2 targeting in *Jak2*V617F MPN settings upon genetic targeting of ERK1/2 in vivo.

### Pharmacologic ERK1/2 inhibition increases susceptibility to JAK2 inhibition in MPN cells

**Fig. 3:**
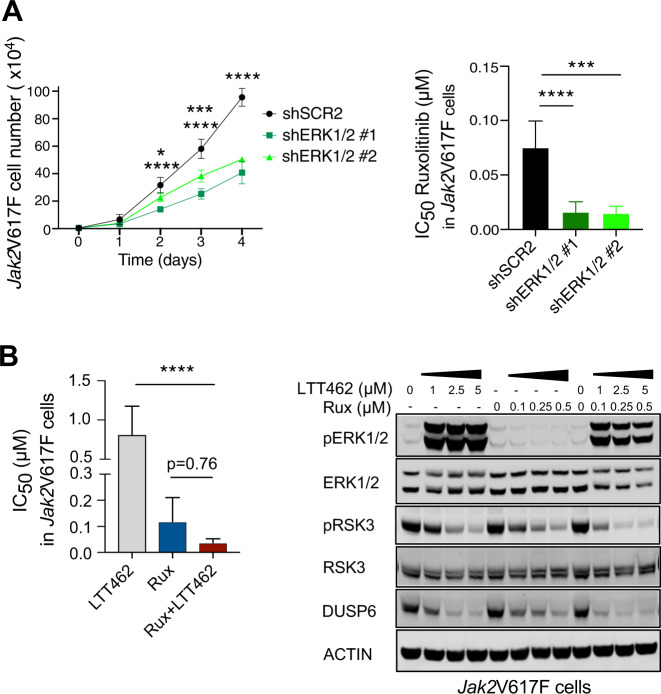
ERK1/2 inhibition increases susceptibility to JAK2 inhibition in *Jak2*V617F cells. **A** Proliferation dynamics of *Jak2*V617F cells was significantly impaired in ERK1/2 deficient settings induced by shRNA-mediated genetic targeting of ERK1/2 with two different hairpins #1 and #2, as indicated by reduced increase of cell count over 4 days (*n* = 3, left panel). ERK1/2 deficient *Jak2*V617F cells were more susceptible to JAK2 inhibition by ruxolitinib at increasing concentrations with 4- to 5-fold reduced half-maximal inhibitory concentration (IC_50_) (*n* = 3, right panel). **B** Pharmacologic ERK1/2 inhibition by LTT462 increased susceptibility of *Jak2*V617F cells to JAK2 inhibition with ruxolitinib with decreased half-maximal inhibitory concentration (IC_50_) (*n* = 3, left panel). Pharmacologic ERK1/2 inhibition by LTT462 dose-dependently suppressed ERK1/2 downstream targets including phosphorylated RSK3 and DUSP6 and enhanced the effects mediated by ruxolitinib when used in combination (*n* = 2, right panel). Data are shown as mean ± SD and were analyzed by one-way ANOVA. *P ≤ 0.05, ***P ≤ 0.001, ****P ≤ 0.0001.

Given these findings suggested enhanced therapeutic efficacy by genetic targeting of ERK1/2, we used a cell-based model to test the potential of novel ERK1/2 inhibitors. In hematopoietic cells expressing *Jak2*V617F along with EPOR, we validated the effects of genetic ERK1/2 targeting observed in vivo, in presence and absence of ruxolitinib. shRNA-mediated ERK1/2 knock-down reduced proliferation dynamics of *Jak2*V617F Ba/F3 cells (Fig. 3A, Supplementary Fig. 6A-B). Similar to ERK1/2 deficiency enhancing effects of JAK2 inhibition in *Jak2*V617F mice, ERK1/2 knock-down increased susceptibility to ruxolitinib in *Jak2*V617F Ba/F3 cells with significantly reduced IC_50_. ERK1/2 downstream targets such as activated RSK3 (pRSK3), DUSP6 and c-MYC expression were reduced most effectively when ruxolitinib and shRNA-mediated ERK1/2 deficiency were combined (Supplementary Fig. 6C-D).

While MEK1/2 inhibitors are used for clinical cancer therapy and have been assessed in MPN models [18, 19], inhibitors targeting ERK1/2 have just been developed recently and have not been explored in MPN settings. We evaluated ERK1/2 inhibitors in *Jak2*V617F Ba/F3 cells for their translational potential including LTT462 and SCH772984, which inhibit ERK activity via ATP-competitive binding [28–30], and DEL-22379 attenuating ERK1/2 dimerization (Fig. 3B, Supplementary Fig. 7) [31]. LTT462 and SCH772984 show differential patterns of ERK1/2 phosphorylation with LTT462 stabilizing and SCH772984 decreasing phospho-ERK1/2 (pERK1/2) [32, 33], while both effectively interfere with ERK1/2 activity and reduce activation of ERK1/2 targets. In *Jak2*V617F cells, LTT462 and SCH772984 enhanced anti-proliferative effects of ruxolitinib resulting in reduced IC_50_ upon combined inhibition without reaching statistical significance (Fig. 3B, *p* = 0.76, Supplementary Fig. 7A-E). Suppression of ERK1/2 targets including pRSK3, DUSP6 and c-MYC expression was more pronounced with dual JAK2/ERK1/2 inhibition as compared to either single agent (Fig. 3B, Supplementary Fig. 7F-G). DEL-22379 did not enhance activity of ruxolitinib and was not pursued further (Supplementary Fig. 7I-M). Dual inhibition of JAK2/ERK1/2 by ruxolitinib/LTT462 enhanced anti-proliferative capacity primarily in *Jak2*V617F cells, whereas *Jak2*WT cells showed less benefit suggesting a potential therapeutic window (Supplementary Fig. 7H, *p* = 0.99). Based on these in vitro profiles, we primarily evaluated LTT462 as ERK1/2 inhibitor in combination with JAK2 inhibition in MPN in vivo settings.

### Dual JAK2 and ERK1/2 inhibition by ruxolitinib/LTT462 enhances therapeutic efficacy in a Jak2V617F MPN preclinical model

**Fig. 4:**
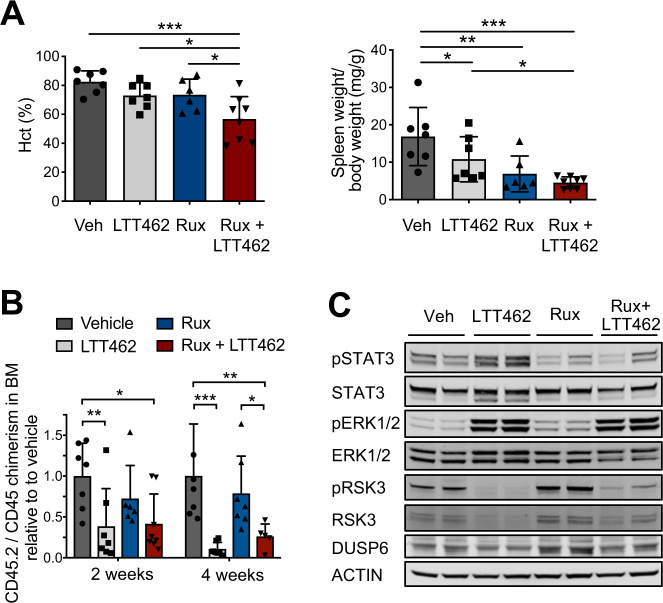
Dual JAK2 and ERK1/2 inhibition by ruxolitinib/LTT462 enhances therapeutic efficacy in a *Jak2*V617F MPN preclinical model. *Jak2*V617F CD45.2 and *Jak2* WT CD45.1 bone marrow (BM) was mixed at 1:1 ratio and transplanted into CD45.1 C57BL/6 recipient mice. Results for 2 weeks of treatment are shown (see Supplementary Fig. 8 for 4 week treatment). **A** Erythrocytosis reflected by increased hematocrit was effectively corrected by combined JAK2/ERK1/2 inhibition with LTT462 at 75 mg/kg qd and ruxolitinib at 60 mg/kg bid (*n* = 6–8/group, left panel). Splenomegaly was moderated by treatment with LTT462 at 75 mg/kg qd or ruxolitinib at 60 mg/kg bid, and LTT462 enhanced ruxolitinib effects when both agents were combined (right panel). **B** Dual ruxolitinib/LTT462 significantly improved control of the *Jak2*V617F clone reflected by CD45.2/CD45 chimerism in BM as compared to ruxolitinib monotherapy. **C** Dual ruxolitinib/LTT462 effectively inhibited the activation of ERK1/2 downstream target RSK3 as shown by reduced phosphorylated RSK3 (pRSK3). Data are presented as mean ± SD. *P ≤ 0.05, **P ≤ 0.01, ***P ≤ 0.001 by one-way ANOVA.

To study the therapeutic potential of dual JAK2 and ERK1/2 inhibition in vivo, we employed an analogous model as for the genetic studies with *Jak2*V617F mutant hematopoiesis alongside *Jak2* wild-type cells via 1:1 BM transplantations. Recipients consistently developed an MPN phenotype with splenomegaly and erythrocytosis as described [18, 23, 25]. Dual inhibition of JAK2 and ERK1/2 by 60 mg/kg ruxolitinib bid and 75 mg/kg LTT462 qd was able to revert splenomegaly within 2 weeks. Combined targeting of JAK2 and ERK1/2 enhanced correction of erythrocytosis as compared to ruxolitinib as a single agent with normalization of Hct, hemoglobin and reticulocytes in the absence of cytopenias (Fig. 4A, Supplementary Fig. 8A-E). Erythroid progenitor populations including Lin^−^Sca1^−^c-Kit^+^CD41^−^FcgRII/III^−^CD150^+^ CD105^−^ megakaryocytic-erythroid progenitors (pre-MegE) and CD71^+^ cells typically expanded in this model [23] were primarily reduced by dual ruxolitinib/LTT462, while ruxolitinib as single agent had more modest effects (Supplementary Fig. 8F-G). *Jak2*V617F mutant hematopoiesis reflected by CD45.2^+^ hematopoietic cells was gradually reduced and clone reductions were evident in erythroid progenitors (Fig. 4B, Supplementary Fig. 8H). We observed as a basis for enhanced phenotype correction that activation of ERK1/2 effectors as RSK3, DUSP6 and ETV5 were significantly reduced by dual inhibition of JAK2 and ERK1/2 as indicated by decreased pRSK3 and significantly lower *Dusp6* and *Etv5* expression. By contrast, these ERK1/2 effectors were not attenuated by ruxolitinib highlighting that ERK1/2 kinase activity needs to be targeted to increase therapeutic efficacy with respect to MAPK signaling (Fig. 4C, Supplementary Fig. 8I). Combined ruxolitinib/LTT462 was tolerable with restoration of BM and spleen architecture and corrective effects were maintained upon prolonged treatment (Supplementary Fig. 8J-S).

### Dual JAK2 and ERK1/2 inhibition by ruxolitinib/LTT462 enhances therapeutic efficacy in a MPLW515L MPN preclinical model

**Fig. 5:**
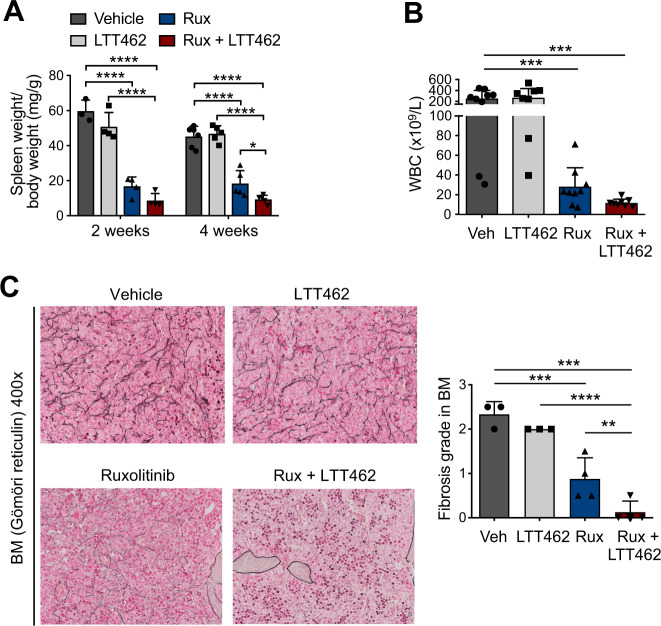
Dual JAK2 and ERK1/2 inhibition by ruxolitinib/LTT462 enhances therapeutic efficacy in a *MPL*W515L MPN preclinical model. **A** Dual JAK2/ERK1/2 inhibition by ruxolitinib at 60 mg/kg bid and LTT462 at 75 mg/kg qd improved splenomegaly control in *MPL*W515L mice (*n* = 3–6/group). **B** Dual JAK2/ERK1/2 inhibition by ruxolitinib/LTT462 enhanced reduction of leukocytosis vs. single agent ruxolitinib at 2 weeks of treatment (*n* = 8–9/group). **C** *MPL*W515L mice consistently developed pronounced BM fibrosis, which was moderated by ruxolitinib and more effectively reduced by combined ruxolitinib/LTT462 at 4 weeks of treatment as shown by reticulin (Gömöri) stain and quantified by a specialized hematopathologist blinded to treatment group assignment (*n* = 3–4/group). Original magnification ×400. Data are shown as mean ± SD and were analyzed by one-way ANOVA. *P ≤ 0.05, **P ≤ 0.01, ***P ≤ 0.001, ****P ≤ 0.0001.

As ERK1/2 deficiency via genetic targeting reduced BM fibrosis, we were interested to explore the potential of pharmacologic ERK1/2 inhibitors to reduce fibrosis in combination with JAK2 inhibitor therapy. We used a *MPL*W515L MPN model characterized by extensive BM fibrosis, splenomegaly, leukocytosis and thrombocytosis, which shares the typical activation of JAK2 and MAPK signaling seen in *Jak2*V617F-driven models [18]. We found that dual JAK2/ERK1/2 inhibition by 60 mg/kg ruxolitinib bid and 75 mg/kg LTT462 qd abrogated splenomegaly and corrected leukocytosis and thrombocytosis (Fig. 5A-B, Supplementary Fig. 9A). BM fibrosis was greatly reduced by combined ruxolitinib/LTT462 and corrective effects were significantly enhanced as compared to ruxolitinib as single agent with consistent decrease of fibrosis by 2 grades (Fig. 5C). Ruxolitinib reduced the expanded BM megakaryocytes as described, while combined ruxolitinib/LTT462 improved correction of splenic architecture and clearance of extramedullary hematopoiesis from the liver and BM cellularity was maintained (Supplementary Fig. 9B-D). We observed that dual JAK2/ERK1/2 inhibition was able to reduce mutant allele burden reflected by GFP^+^ cells in blood, BM and spleen with consistent clone reductions in animals on combination treatment and that dual inhibition was able to prolong survival as compared to vehicle-treated animals (Supplementary Fig. 9E-I). Corrective effects were maintained upon extended treatment despite the aggressive disease dynamics. Thrombocytopenia, which progressively developed in the longer course limited survival benefit in this model (Supplementary Fig. 9J-N). Analyses of serum concentrations in steady state excluded accumulation of inhibitors when administered as combination in *MPL*W515L or *Jak2*V617F settings suggesting combining JAK2 and ERK1/2 inhibition was safe (Supplementary Fig. 10).

### Enhanced therapeutic efficacy of dual JAK2/ERK1/2 inhibition in Jak2V617F and MPLW515L MPN models is confirmed with the alternative ERK1/2 inhibitor MK-8353

Given ERK1/2 inhibitors have recently emerged and compounds with differential profiles are in development, we sought to validate the enhanced corrective potential of dual JAK2/ERK1/2 inhibition using a second compound. We employed MK-8353, an ATP-competitive ERK1/2 inhibitor derived from SCH772984 with refined pharmacologic properties suitable for in vivo use [29, 30, 32, 33]. In addition to interfering with ERK1/2 activity via ATP-competitive binding, MK-8353 interfered with phosphorylation of ERK1/2 by MEK1/2 as described [32, 33] (Supplementary Fig. 11A). Similar to ruxolitinib/LTT462, dual JAK2/ERK1/2 inhibition by 60 mg/kg ruxolitinib bid and 30 mg/kg MK-8353 bid enhanced MPN phenotype correction in the *MPL*W515L model including reduction of splenomegaly, leukocytosis and thrombocytosis. Ruxolitinib/MK-8353 was able to reduce BM fibrosis reflected by reticulin stain along with consistent albeit subtle reduction of mutant allele burden and improved survival (Supplementary Fig. 11B-K). Also in *Jak2*V617F settings, ruxolitinib/MK-8353 showed comparable effects in normalizing erythrocytosis and splenomegaly as seen with ruxolitinib/LTT462 (Supplementary Fig. 12A-H), thus credentialing combined JAK2/ERK1/2 inhibition as a valid approach to enhance the corrective potential of JAK2 inhibitor therapy for MPN.

### Dual JAK2 and ERK1/2 inhibition by ruxolitinib/LTT462 enhances suppression of myeloid colony outgrowth and ERK1/2 target activation from primary JAK2V617F patient cells

**Fig. 6:**
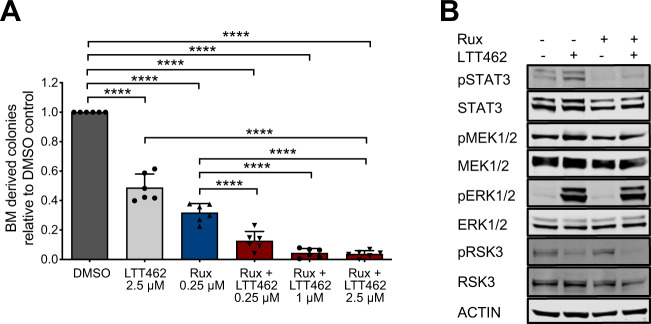
Dual JAK2 and ERK1/2 inhibition enhances suppression of myeloid colony formation and ERK1/2 target activation from primary *JAK2*V617F patient cells. Paired bone marrow (BM) and blood isolates from *JAK2*V617F mutated MPN patients were assessed for the potential of myeloid colony outgrowth upon exposure to dual JAK2 and ERK1/2 inhibition by ruxolitinib/LTT462. Whole BM cells were seeded at 150.000 cells/well and scored after 10 days, while peripheral blood mononuclear cells (PBMCs) were enriched for CD34^+^ cells and seeded into methocult at 3000 cells/well (see Supplementary Fig. 13). **A** ERK1/2 inhibition by LTT462 at 0.25, 1 and 2.5 µM improved control of myeloid colony formation from BM cells seen with ruxolitinib at 0.25 µM in a dose-dependent manner (*n* = 6). **B** Analysis of signaling in freshly isolated PBMCs from a *JAK2*V617F mutated MPN patient exposed to inhibitors ex vivo for 16 h showed improved inhibition of ERK1/2 downstream target RSK by ruxolitinib/LTT462 as reflected by reduced RSK3 phosphorylation (pRSK3). Data are presented as mean ± SD. ***P ≤ 0.001, ****P ≤ 0.0001 by one-way ANOVA.

To evaluate the corrective potential of combined JAK2 and ERK1/2 inhibition in human MPN settings, we tested clinical isolates from JAK2V617F MPN patients including PMF, PV and ET. Paired blood and BM samples were from initial diagnosis in the absence of cytoreductive therapies (Supplementary Fig. 13A). CD34^+^ enriched peripheral blood mononuclear cells (PBMC) as well as whole BM cells seeded into methylcellulose were exposed to 0.25 μM ruxolitinib and/or LTT462 at increasing concentrations to assess the impact of dual JAK2/ERK1/2 inhibition on myeloid colony formation. Overall, we observed that ERK1/2 inhibition moderated outgrowth of colonies from CD34^+^ and BM cells in the absence of JAK2 inhibition similar to the findings in ERK1/2-deficient *Jak2*V617F murine models. Most importantly, ERK1/2 inhibition enhanced JAK2 inhibitor efficacy dose-dependently with gradually reduced colony numbers in presence of ruxolitinib when LTT462 concentrations were further increased, indicating an additional corrective effect of ERK1/2 inhibition in human MPN settings (Fig. 6A, Supplementary Fig. 13B). Enhanced suppression of colony outgrowth was not restricted to a specific subtype of MPN, but seen in primary cells from PMF, PV or ET patients (Supplementary Fig. 13C). Erythroid as well as granulocytic-macrophage colony subtypes were affected by enhanced ruxolitinib/LTT462 effects (Supplementary Fig. 13D). Phosphorylated RSK3 reflecting ERK1/2 target activation was more thoroughly suppressed by dual JAK2/ERK1/2 inhibition than with ruxolitinib as single agent suggesting that impeding ERK1/2 activity can enforce efficacy of JAK2 inhibitor treatment in JAK2V617F human cells (Fig. 6B). For confirmation, CD34^+^ cells from MPL-mutated MPN patients were also assessed showing analogous effects upon combined JAK2/ERK1/2 inhibition to *JAK2*V617F patients, while CD34^+^ cells from healthy donors were evaluated as controls (Supplementary Fig. 13E-J).

## Discussion

Constitutive activation of the JAK2 signaling network is a hallmark of MPN and has led to the clinical use of JAK2 inhibitors as standard treatment in MF and refractory PV [3, 34–36]. JAK2 inhibitor therapy holds benefits particularly in regard to splenomegaly and symptom control, but it has become clear that disease-modifying activity is limited with continued clonal evolution and responses restricted to finite periods [37–39]. Therefore, it is imperative to uncover molecular mechanisms impeding JAK2 inhibitor efficacy, which can inform rationally designed therapeutic approaches with enhanced corrective potential. The MAPK pathway with the sequential kinases RAF, MEK and ERK is known to be involved in several cancers including leukemias [9, 10]. There is increasing insight into MAPK pathway activation in MPN, particularly upon JAK2 inhibitor treatment. Cell-extrinsic and intrinsic mechanisms have been reported, which unanimously highlight the significance of MAPK activation for the limited efficacy of JAK2 inhibitors [18–20]. BRAF and MEK inhibitors, which are in use for treatment of other malignancies, have allowed to validate the relevance of MAPK signaling in MPN [13, 14, 18, 19]. Additional therapeutic benefit was observed in preclinical models upon treatment with the MEK inhibitors binimetinib, selumetinib [18], trametinib and PD0325901 [19] when combined with JAK2 inhibition.

Given the significant role of MAPK pathway signaling in impeding JAK2 inhibitor efficacy in MPN, we hypothesized that direct inhibition of ERK1/2 kinases, for which inhibitors have recently become available and have not been specifically explored in MPN, could represent a beneficial therapeutic approach for several reasons: (1) Targeting at the more distal node of ERK1/2 in the MAPK pathway could reduce adaptive signaling changes and reduce the potential for escape from combined JAK2/ERK1/2 inhibition [7]. (2) ERK1/2 are directly upstream of an array of MAPK pathway effector molecules, allocating ERK1/2 immediate control of MAPK pathway-triggered transcriptional programs [8]. (3) Most importantly, it has been shown that ERK1/2 exert essential roles for hematopoiesis with ERK1/2 loss mediating BM aplasia in non-diseased hematopoietic settings [21, 22]. Here we employed genetic and pharmacologic approaches to evaluate dual targeting of JAK2 and ERK1/2 as a therapeutic approach in MPN. We evaluated corrective effects in MPN preclinical models, cells and patient clinical isolates. Genetic targeting of ERK1/2 mitigated the MPN phenotype in *Jak2*V617F mice including clone reductions highlighting a dependency of MPN cells on ERK1/2 for proliferation and survival. ERK1/2 deficiency significantly enhanced therapeutic efficacy of concomitant JAK2 inhibitor treatment regarding erythrocytosis, splenomegaly, fibrosis and clone control. Similarly, dual targeting of JAK2 and ERK1/2 using novel ATP-competitive ERK1/2 inhibitors LTT462 and MK-8353 in combination with ruxolitinib enhanced therapeutic performance in *Jak2*V617F mice and was effective in the more aggressive *MPL*W515L model including potent fibrosis-reducing effects. An alternative ERK1/2 inhibitor, DEL-22379, interfering with ERK1/2 dimerization, did not induce substantial additional benefit in combination with ruxolitinib. This difference in efficacy may relate to the differential mechanism of action with the ERK dimerization inhibitor impacting on cytoplasmic but not nuclear ERK targets, whereas ATP-competitive ERK inhibitors seem to act more broadly at a generally high specificity [31, 40]. Although pharmacologic JAK2/ERK1/2 inhibition does not have specific mutant-selective properties, we saw an influence on the MPN clone with inhibitor treatment, which in line with the genetic model suggests a potential for clone control. Given the essential role of ERK1/2 for hematopoiesis [21, 22], specific vulnerabilities of *JAK2*V617F mutant and wild-type settings to ERK1/2 inhibition should be explored and dedicated investigations of applicable dosages and treatment schedules will be important as part of initial clinical studies. Our studies in paired blood and BM isolates from *JAK2*V617F MPN patients provide first evidence of enhanced corrective effects of dual JAK2/ERK1/2 inhibition as compared to ruxolitinib along with improved ERK1/2 target suppression in patient cells. Overall, our data demonstrates that dual targeting of JAK2 and ERK1/2 effectively addresses ERK1/2 kinases as a second node of oncogenic signaling, which warrants inhibition in MPN. We show here that dual targeting of JAK2 and ERK1/2 leads to an enhanced therapeutic performance in several MPN settings and thus should be pursued as a mechanism-based therapeutic approach for MPN patients.

## Supplementary information

Supplemental Methods and Figures
