# Supplementary material for: Dual targeting of JAK2 and ERK interferes with the myeloproliferative neoplasm clone and enhances therapeutic efficacy
Source: Leukemia. 2021 Sep 3;35(10):2875–84. doi: 10.1038/s41375-021-01391-2 (PMC8478661; doi:10.1038/s41375-021-01391-2)
Supplement: Supplementary file 1 — Supplemental Methods and Figures [file 41375_2021_1391_MOESM1_ESM.pdf]

Supplemental information to "Dual targeting of JAK2 and ERK interferes with the myeloproliferative neoplasm clone and enhances therapeutic efficacy"

Sime Brkic[1*], Simona Stivala[1*], Alice Santopolo[1], Jakub Szybinski[1], Sarah Jungius[1], Jakob R. Passweg[2], Dimitrios Tsakiris[2], Stefan Dirnhofer[3], Gregor Hutter[1], Katharina Leonards[2], Heidi E.L. Lischer[4,5], Matthias Dettmer[4], Benjamin G. Neel[5], Ross L. Levine[6], Sara C. Meyer[1,2]

[1]Department of Biomedicine, University Hospital Basel and University of Basel, Basel, Switzerland; [2]Division of Hematology, University Hospital Basel, Basel, Switzerland; [3]Department of Pathology, University Hospital Basel, Basel, Switzerland; [4]Interfaculty Bioinformatics Unit, University of Bern, Bern, Switzerland; [5]Swiss Institute of Bioinformatics, Lausanne, Switzerland; [6]Department of Pathology, University of Berne, Bern, Switzerland; [7]Laura and Isaac Perlmutter Cancer Center, New York University Langone, New York, NY, USA; [8]Human Oncology and Pathogenesis Program and Leukemia service, Memorial Sloan Kettering Cancer Center, New York, NY, USA; *equal contribution (alphabetical order)

Supplemental Methods

Flow cytometry. Antibodies used for flow cytometry were directed to mouse Sca-1 (clone D7), c-Kit (clone 2B8), CD41 (clone MWReg30), CD150 (clone TC15-12F12.2), CD48 (clone HM48-1), CD71 (clone RI7217), Ter119 (clone TER-119), CD45.1 (clone A20), CD45.2 (clone 104) from Biolegend and CD16/CD32 (clone 93) and CD105 (clone MJ7/1B) from eBiosciences.

Signaling analyses. Antibodies used for signaling analyses in cell lines, mouse models and patient samples included pERK1/2 (Clone D13.14.4E, Cat.No. 4370), ERK1/2 (Clone 137F5, Cat.No. 4695), pMEK1/2 (Clone 41G9, Cat.No. 9154), MEK1/2 (Clone 47E6, Cat.No. 9126), pRSK3 (Cat.No. 9348), pc-MYC (CloneE1J4K, Cat.No.13784), c-MYC (Clone D84C12, Cat.No. 5605), pSTAT3 (Clone D3A7, Cat.No. 9145), STAT3 (Clone 79D7, Cat.No. 4904), pSTAT5 (Clone C11C5, Cat.No. 9359), STAT5 (Clone D206Y, Cat.No. 94205), actin (Clone 8H10D10, Cat.No.3700) and GAPDH (Clone D4C6R, Cat.No. 97166) from Cell Signaling Technology, and RSK3 (3C4C8, Cat.No. sc-517283) and DUSP6 (F-12, Cat.No. sc-377070) from Santa Cruz Biotechnology. Image Studio software (Li-Cor Biosciences) was used for densitometries of band intensities.

RNA expression analysis by Nanostring. Raw Nanostring counts were normalized using the hierarchical modeling approach of the NanoStringDiff R package incorporating 6 housekeeping, 6 positive control and 8 negative control genes to correct for variation in input sample material, lane-by-lane variation and non-specific background noise, respectively (Hong Wang, Tingting Zhai and Chi Wang (2019). NanoStringDiff: Differential Expression Analysis of NanoString nCounter Data. R package version 1.16.0., R version 3.6.2). Complete information from NanoString nCounter Analyzer was used for the normalization process. NanoStringDiff R package was also used to obtain differentially expressed genes based on a generalized linear model of the negative binomial family and a Benjamini and Hochberg procedure for multiple testing correction. Heatmaps were created using ComplexHeatmap R package to show z-score transformed log2 normalized gene expression counts(17). Principal component analysis (PCA) was based on log2 transformed normalized gene expression counts using prcomp function of R.

Mutational analysis of MPN patients by next generation sequencing. Mutational status was assessed using a 39-gene targeted AmpliSeq NGS assay covering full genes including splice sites including ABL1, ASXL1, BCR, BRAF, CALR, CBL, CEBPA, CHEK2, CSF3R, DNMT3A, EGLN1, EPOR, ETNK1, ETV6, EZH2, FLT3, GATA2, IDH1, IDH2, JAK2, KIT, KRAS, MPL, NF1, NPM1, NRAS, PDGFRA, PDGFRB, PTPN11, RUNX1, SETBP1, SF3B1, SH2B3, SRSF2, TET2, TP53, U2AF1, VHL, ZRSR2. A total of 60 ng DNA was amplified with 15 PCR

cycles during automated library preparation with the Ion AmpliSeq Kit for Chef DL8 (Cat. A29024) on the IonChef instrument. Library pools were quantified by qPCR using the Ion Universal Quantitation Kit (Cat. A26217) and 30 pmol of the pool was sequenced on 530 Chips on an Ion S5XL sequencer. Sequences were aligned to the human reference genome GRCh37/hg19 and analyzed with IonReporter version 5.12 with a sensitivity limit of variant allelic frequency of 2-5%.

Supplemental Tables

Supplemental Table 1. qPCR primer sequences.

Target	Primer sequence
Erk1	FW: 5'-GCAGTCTCTGCCCTCGAAAA -3'
	REV: 5'-AGGTCAAGAGCTTTGGAGTCA -3'
Erk2	FW: 5'- GTGATCTCAAGATCTGTGACTTTGG-3'
	REV: 5'-CGTGTGGCTACGTACTCTGT -3'
Etv5	FW: 5'-GTCCTCCAGCACATGGGTTC-3'
	REV: 5'-GGCAGTTAGGCACTTCTGAGT-3'
Dusp6	FW: 5'-GTTCTACCTGGAAGGTGGCT-3'
	REV: 5'-CTGCACGAGCCGTCTAGATT-3'
Gapdh	FW: 5'-CGTCCCGTAGACAAAATGGT-3'
	REV: 5'-TTGATGGCAACAATCTCCAC-3'

Supplemental Table 2. shRNA target sequences.

Target	shRNA	Target sequence
Control	shSCR2	5'-CAACAAGATGAAGAGCACCAA-3'
*Erk*1/2	shERK1/2 #1	5'-AGCAATGACCACATCTGCTA-3'
	shERK1/2 #2	5'-CAGGACCTCATGGAGAC-3'

Supplemental Figure 1

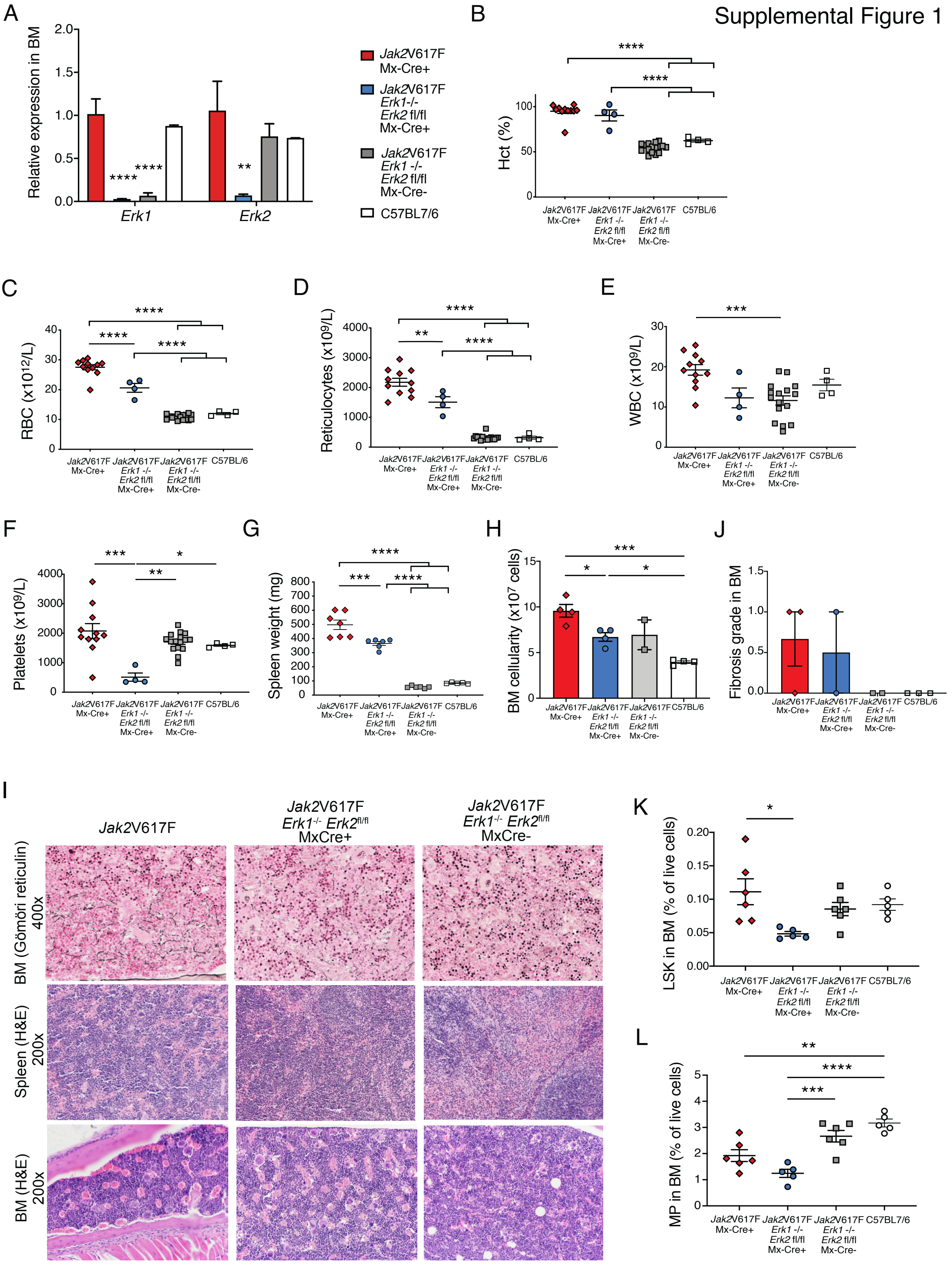

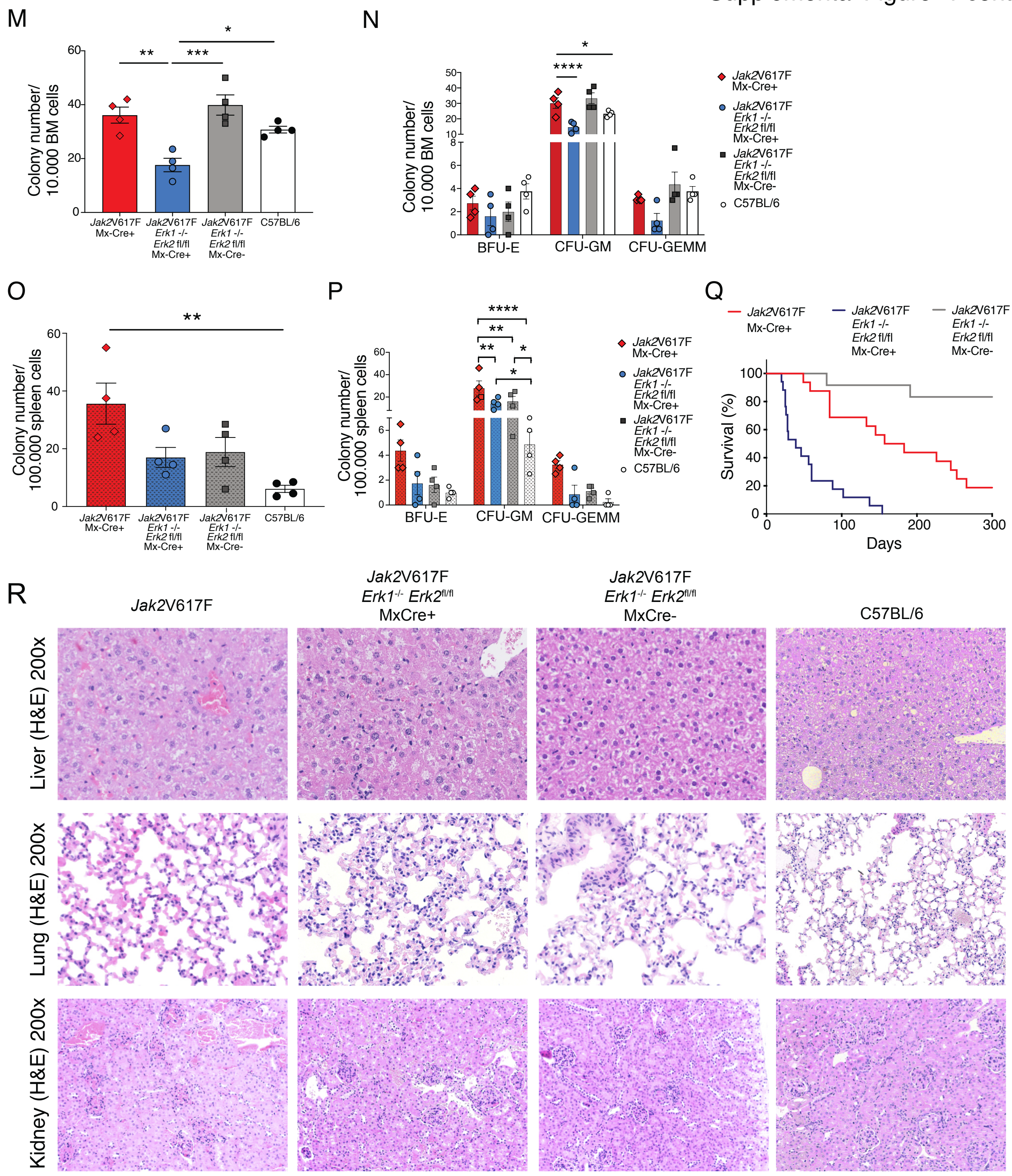

Supplemental Figure 1. Genetic targeting of ERK1/2 mitigates the MPN phenotype of *Jak2*V617F primary mice. (**A**) Conditional *Jak2*V617F knock-in mice were crossed to *Erk1*$^{-/-}$*Erk2*$^{f/f}$ mice expressing Mx-1-Cre recombinase to generate *Jak2*V617F *Erk1*$^{-/-}$ *Erk2*$^{f/f}$ Mx-Cre^{+} mice. ERK1/2 expression was assessed in bone marrow (BM) 1 month after poly I:C (pIpC) induction. Expression relative to *Jak2*V617F Mx-Cre^{+} mice is indicated showing significant ERK1/2 deficiency in ERK1/2-

targeted mice (n=4-5/group, except for C57BL/6 controls n=3). (**B**) Hematocrit, (**C**) red blood cells (RBC), (**D**) reticulocytes, (**E**) white blood cells (WBC) and (**F**) platelets in ERK1/2-deficient *Jak2*V617F mice 2 months after pIpC induction were significantly moderated as compared to *Jak2*V617F mice with intact ERK1/2 (n=4-16/group). (**G**) Splenomegaly was significantly mitigated in ERK1/2-deficient mice 1 month after pIpC induction (n=5-7/group). (**H**) BM cellularity was lower in ERK1/2-deficient *Jak2*V617F mice as compared to *Jak2*V617F mice with intact ERK1/2 and equivalent to Mx-Cre-negative control mice (n=2-4/group). (**I-J**) BM fibrosis as shown by reticulin (Gömöri) stain was reduced in ERK1/2-deficient *Jak2*V617F mice as compared to *Jak2*V617F mice with intact ERK1/2 1 month after pIpC induction. Original magnification 400x. Spleen architecture, which was effaced in *Jak2*V617F mice, was partially restored upon ERK1/2 deficiency. Original magnification 200x (n=2-3/group). (**K-L**) Quantification of BM Lin$^-$Sca1$^+$Kit$^+$ (LSK) and Lin$^-$Sca1$^-$Kit$^+$ multipotent myeloid progenitors (MP) showed a decrease in ERK1/2-deficient *Jak2*V617F mice as compared to *Jak2*V617F mice with intact ERK1/2 1 month after pIpC induction (n=5-6/group). (**M-N**) ERK1/2 deficiency in *Jak2*V617F mice resulted in a significantly reduced colony number from BM cells including erythroid (BFU-E), granulocytic-macrophage (CFU-GM) and granulocyte-erythroid-macrophage-megakaryocyte (CFU-GEMM) subtypes 1 month after pIpC (n= 4/group). (**O-P**) ERK1/2 deficiency in *Jak2*V617F mice resulted in a significantly reduced colony number from spleen cells including erythroid (BFU-E), granulocytic-macrophage (CFU-GM) and granulocyte-erythroid-macrophage-megakaryocyte (CFU-GEMM) subtypes (n=4/group). (**Q**) Kaplan-Meyer survival analysis showed reduced survival of *Jak2*V617F mice with intact and more pronounced with deficient ERK1/2 (n=12-17/group). (**R**) Representative images of liver, lung and kidney of *Jak2*V617F mice with intact and deficient ERK1/2 showed normal organ architecture as compared to Mx-Cre-negative control mice. Original magnification 200x. Data shown as mean ± SEM and analyzed by one-way ANOVA. *P ≤ 0.05, **P ≤ 0.01, ***P ≤ 0.001, ****P ≤ 0.0001.

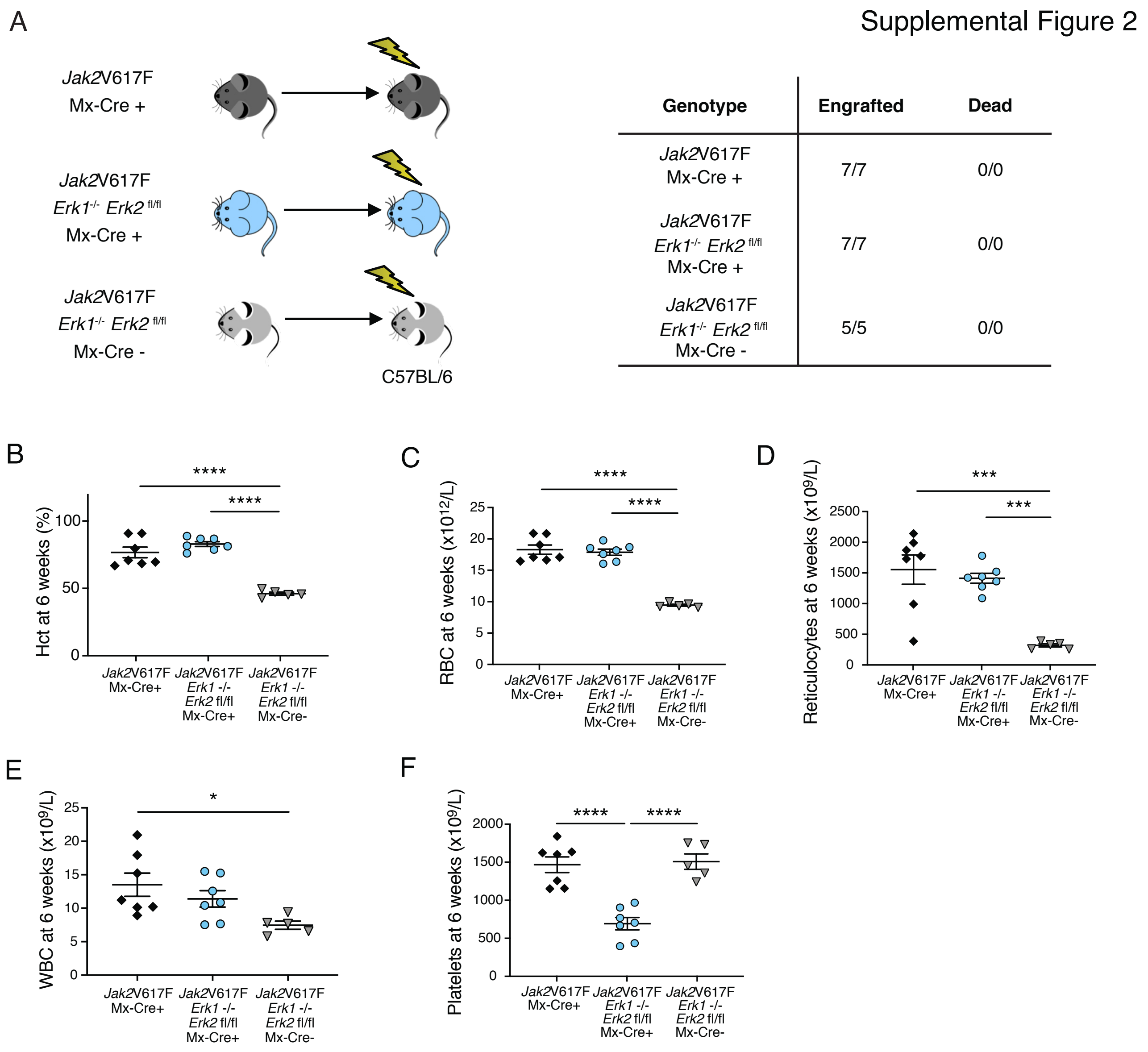

Supplemental Figure 2. Genetic targeting of ERK1/2 does not impair engraftment potential of *Jak2*V617F bone marrow. (**A**) Schema of non-competitive bone marrow (BM) transplantation setup. Whole BM cells from *Jak2*V617F mice with intact or deficient ERK1/2 as well as from Mx-Cre-negative control mice consistently engrafted in lethally irradiated BL/6 recipient mice as indicated in the table (n=5-7/group). (**B**) Hematocrit, (**C**) red blood cells (RBC), (**D**) reticulocytes, (**E**) white blood cells (WBC) and (**F**) platelets 6 weeks after transplantation are shown indicating consistent trilineage engraftment and regeneration (n=5-7/group). Data shown as mean ± SEM and analyzed by one-way ANOVA. *P ≤ 0.05, ***P ≤ 0.001, ****P ≤ 0.0001.

Supplemental Figure 3

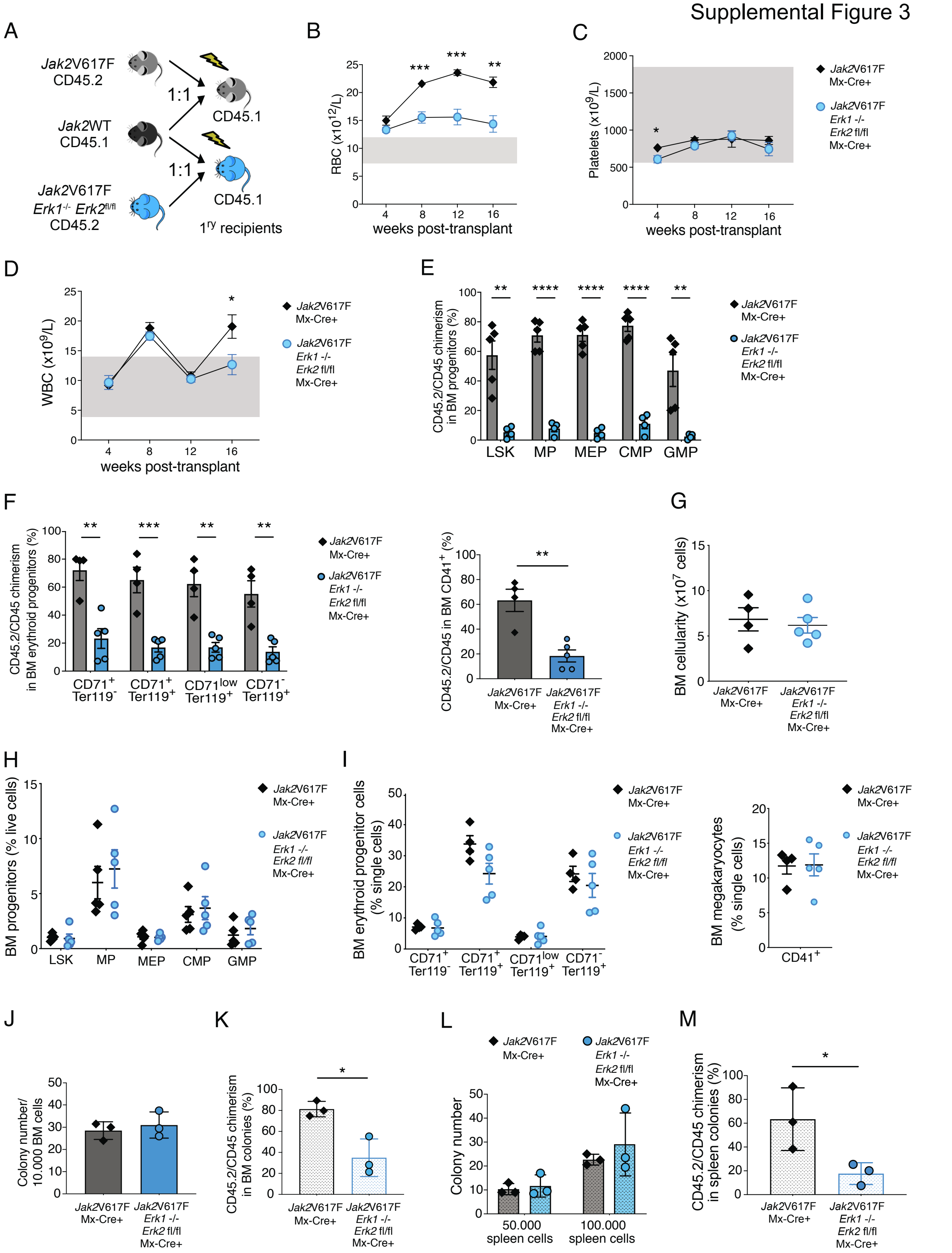

Supplemental Figure 3 cont.

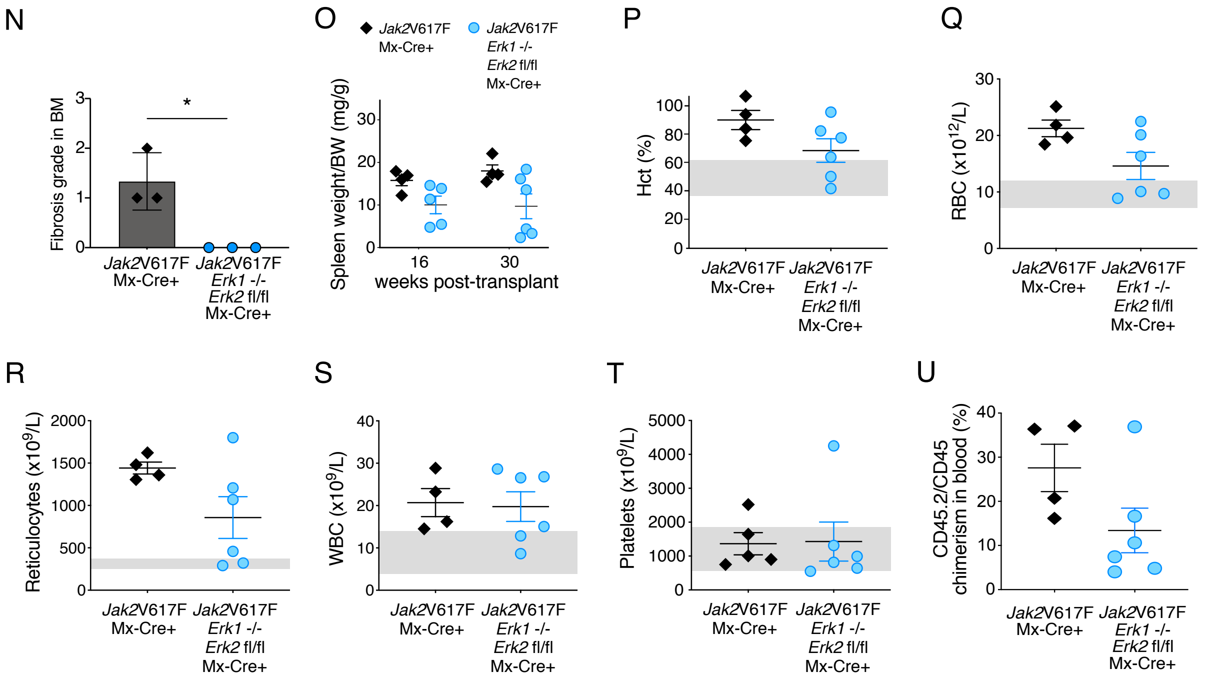

Supplemental Figure 3. Genetic targeting of ERK1/2 mitigates the MPN phenotype and impairs the *Jak2*V617F clone upon competitive *Jak2*V617F / *Jak2*WT bone marrow transplantation (additional data to Figure 1). (**A**) Schema of transplantation model with *Jak2*V617F CD45.2 bone marrow (BM) with intact (grey) or deficient (blue) ERK1/2 in 1:1 ratio with *Jak2*WT CD45.1 BM (black) into CD45.1 C57BL/6 recipient mice. (**B**) Erythrocytosis reflected by red blood cell count was moderated by ERK1/2 deficiency in *Jak2*V617F settings (n=10-11/group, shaded areas represent normal range). (**C**) Platelets were not lowered by ERK1/2 deficiency in this *Jak2*V617F model, and (**D**) leukocytes remained in the normal range (n=10-11/group). (**E**) CD45.2/CD45 chimerism was significantly reduced by ERK1/2 deficiency in BM stem/progenitor populations including Lin$^-$Sca1$^+$Kit$^+$ (LSK), Lin$^-$Sca1$^-$Kit$^+$ multipotent myeloid (MP), Lin$^-$Sca1$^-$c-Kit$^+$FcgR^{low}CD34$^-$ megakaryocyte-erythroid (MEP), Lin$^-$Sca1$^-$c-Kit$^+$FcgR^{low}CD34$^+$ common myeloid (CMP) and Lin$^-$Sca1$^-$c-Kit$^+$FcgR^{high}CD34$^+$ granulocyte-monocyte (GMP) progenitor compartments. (**F**) BM erythroid progenitor cells as well as megakaryocytic cell populations showed significant reduction of CD45.2/CD45 chimerism (n=4-5/group). (**G**) Recipients of *Jak2*V617F or *Jak2*V617F *Erk1*$^{-/-}$ *Erk2*$^{fl/fl}$ BM cells mixed 1:1 with *Jak2*WT cells showed analogous BM cellularity 16 weeks after transplantation. (**H**) Frequencies of BM myeloid stem/progenitor populations including Lin$^-$Sca1$^+$Kit$^+$ (LSK), Lin$^-$Sca1$^-$Kit$^+$ multipotent myeloid (MP), Lin$^-$Sca1$^-$c-Kit$^+$FcgR^{low}CD34$^-$ megakaryocyte-erythroid (MEP), Lin$^-$Sca1$^-$c-Kit$^+$FcgR^{low}CD34$^+$ common myeloid (CMP) and Lin$^-$Sca1$^-$c-Kit$^+$FcgR^{high}CD34$^+$ granulocyte-monocyte (GMP) progenitor compartments were not different in recipients of *Jak2*V617F BM with intact or deficient ERK1/2. (**I**) Frequencies of BM erythroid progenitor cells as well as of megakaryocytic cells were not different in recipients of *Jak2*V617F BM with intact or deficient ERK1/2. (**J**) Colony-forming potential from BM of competitively transplanted mice was unaffected by ERK1/2 deficiency. (**K**) Contribution from the *Jak2*V617F clone to emerging colonies was significantly reduced upon ERK1/2 deficiency as indicated by CD45.2/CD45 chimerism

(n=3/group). (**L**) Colony-forming potential from spleen of competitively transplanted mice was not affected by ERK1/2 deficiency. (**M**) Contribution from the *Jak2*V617F clone to emerging colonies was significantly reduced upon ERK1/2 deficiency as indicated by CD45.2/CD45 chimerism (n=3/group). (**N**) BM fibrosis assessed 30 weeks after transplantation was not evident in ERK1/2 deficient *Jak2*V617F mice, whereas fibrosis was detected in Jak2V617F mice with intact ERK1/2 as reflected by fibrosis grading by a specialized hematopathologist blinded for the respective genotypes (n=3/group). (**O**) ERK1/2 deficiency reduced splenomegaly at 16 and 30 weeks after transplantation. (**P-U**) Blood counts and peripheral blood CD45.2/CD45 chimerism indicating *Jak2*V617F mutant allele burden showed maintained effects of ERK1/2 deficiency 30 weeks after transplantation. Data shown as mean ± SEM and analyzed by Student *t*-test. *P ≤ 0.05, **P ≤ 0.01, ***P ≤ 0.001, ****P ≤ 0.0001.

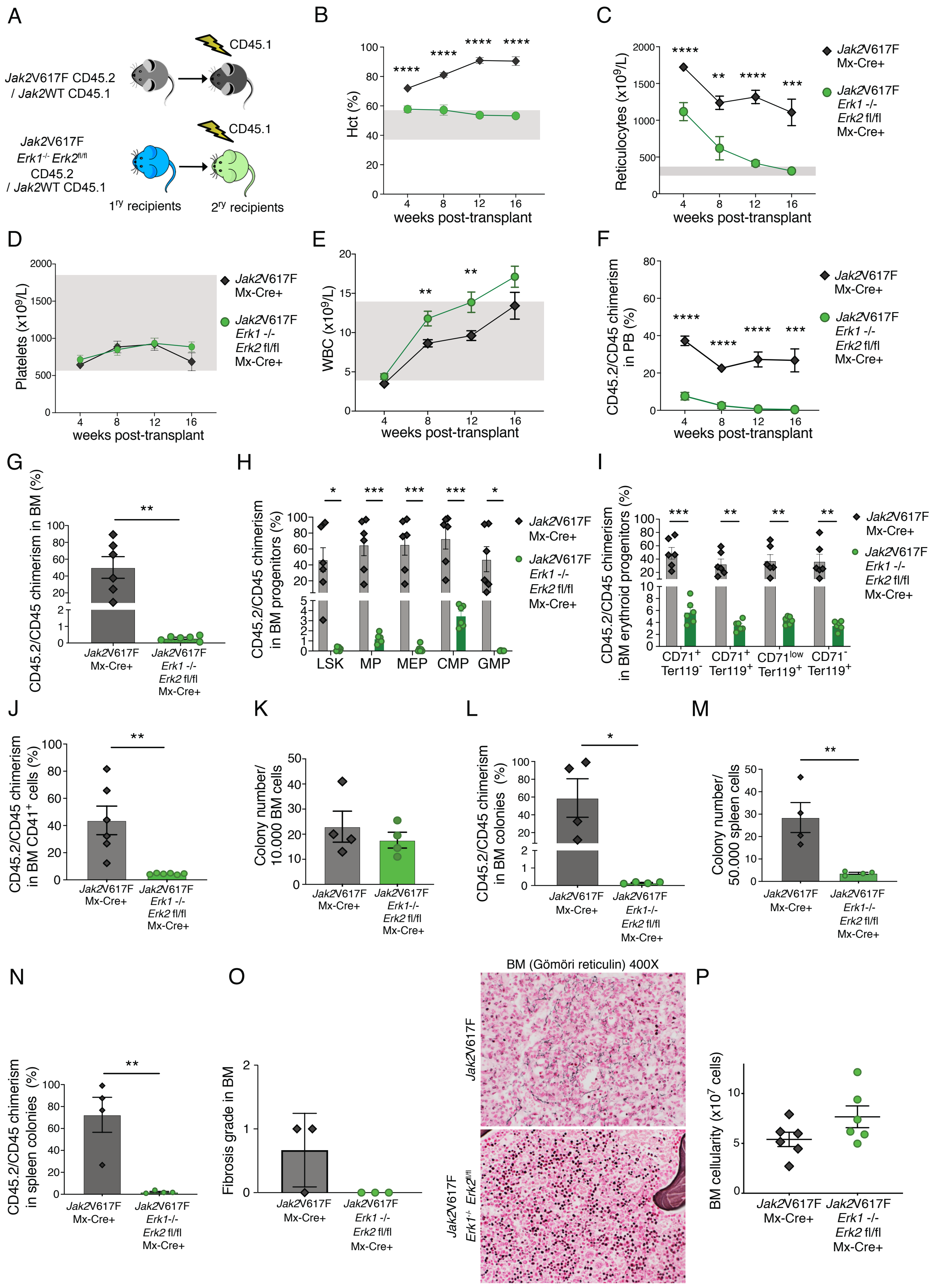
A
Jak2V617F CD45.2 / Jak2WT CD45.1
Jak2V617F Erk1-/- Erk2fl/fl CD45.2 / Jak2WT CD45.1
CD45.1
1ry recipients
2ry recipients
B
Hct (%)
weeks post-transplant
C
Reticulocytes (x10^9/L)
D
Platelets (x10^9/L)
E
WBC (x10^9/L)
F
CD45.2/CD45 chimerism in PB (%)
Jak2V617F Mx-Cre+
Jak2V617F Erk1 -/- Erk2 fl/fl Mx-Cre+
G
CD45.2/CD45 chimerism in BM (%)
H
CD45.2/CD45 chimerism in BM progenitors (%)
LSK MP MEP CMP GMP
I
CD45.2/CD45 chimerism in BM erythroid progenitors (%)
CD71+ Ter119- CD71+ Ter119+ CD71low Ter119+ CD71- Ter119+
J
CD45.2/CD45 chimerism in BM CD41+ cells (%)
K
Colony number/ 10.000 BM cells
L
CD45.2/CD45 chimerism in BM colonies (%)
M
Colony number/ 50.000 spleen cells
N
CD45.2/CD45 chimerism in spleen colonies (%)
O
Fibrosis grade in BM
BM (Gömöri reticulin) 400X
Jak2V617F
Jak2V617F Erk1-/- Erk2fl/fl
P
BM cellularity (x10^7 cells)

Supplemental Figure 4 cont.

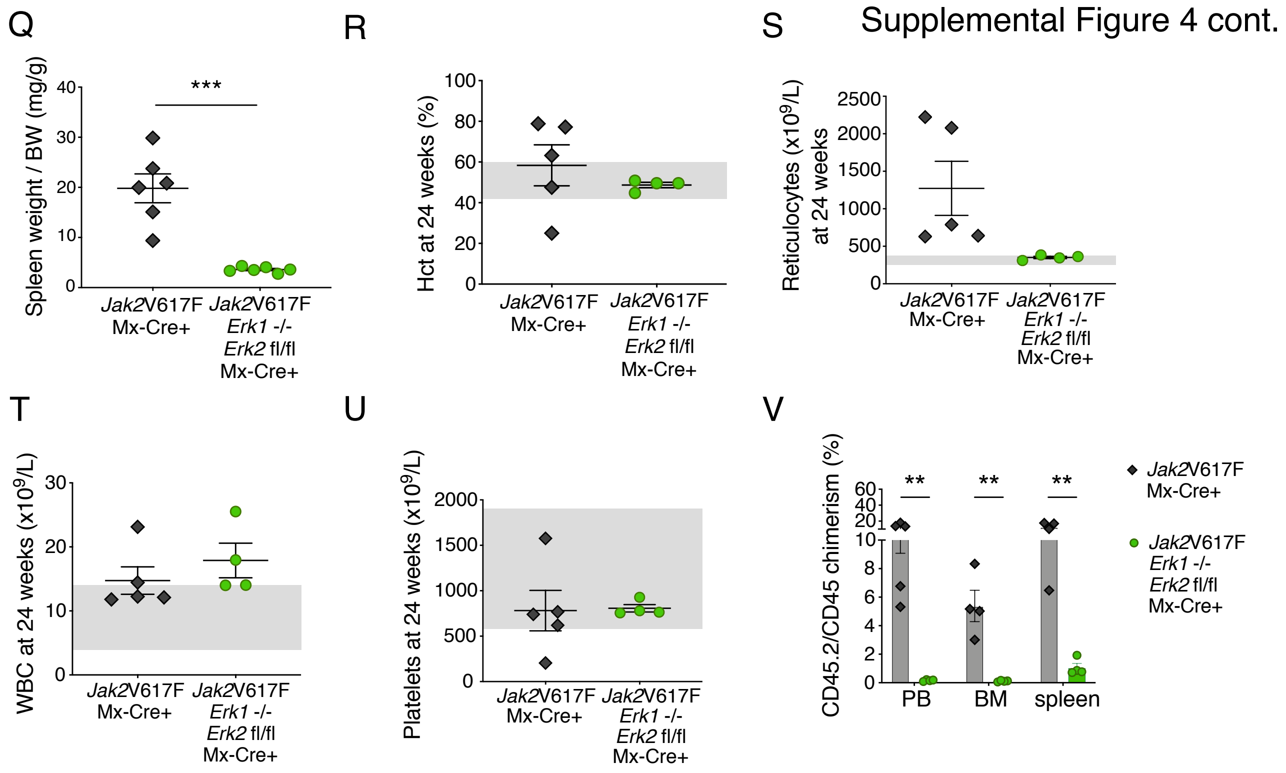

Supplemental Figure 4. Effects of genetic ERK1/2 targeting are maintained upon secondary transplantation in *Jak2*V617F mice. (**A**) Schema of secondary transplantations of BM from primary recipients, which had received *Jak2*V617F / *Jak2*WT competitive transplantations with intact (grey) or deficient (blue) ERK1/2 (see Figure 1) into secondary CD45.1 BL/6 recipient mice (dark grey: intact ERK1/2, green: deficient ERK1/2). (**B**) Erythrocytosis reflected by increased hematocrit and (**C**) reticulocytes was corrected by ERK1/2 deficiency in *Jak2*V617F settings. (**D**) Platelets and (**E**) leukocytes were not relevantly lowered by ERK1/2 deficiency (n=11-12/group, shaded areas represent normal range). (**F**) CD45.2/CD45 chimerism reflecting *Jak2*V617F allele burden was significantly reduced by ERK1/2 deficiency in peripheral blood (n=11-12/group) and (**G**) bone marrow (BM) 16 weeks after secondary transplantation (n=6/group). (**H**) CD45.2/CD45 chimerism in secondary recipients was significantly reduced by ERK1/2 deficiency also in BM stem/progenitor populations including Lin$^-$Sca1$^+$Kit$^+$ (LSK), Lin$^-$Sca1$^-$Kit$^+$ multipotent myeloid (MP), Lin$^-$Sca1$^-$c-Kit$^+$FcgR^{low}CD34$^-$ megakaryocyte-erythroid (MEP), Lin$^-$Sca1$^-$c-Kit$^+$FcgR^{low}CD34$^+$ common myeloid (CMP) and Lin$^-$Sca1$^-$c-Kit$^+$FcgR^{high}CD34$^+$ granulocyte-monocyte (GMP) progenitors, (**I**) erythroid progenitors as well as (**J**) megakaryocytic cells (n=6/group). (**K**) Colony-forming potential from BM of secondary recipient mice was not significantly affected by ERK1/2 deficiency. (**L**) Contribution from the *Jak2*V617F clone to emerging colonies was significantly reduced by ERK1/2 deficiency as indicated by CD45.2/CD45 chimerism (n=4/group). (**M**) Colony-forming potential from spleen of secondary recipient mice as well as (**N**) contribution from the *Jak2*V617F clone was significantly reduced upon ERK1/2 deficiency as indicated by CD45.2/CD45 chimerism (n=4/group). (**O**) BM fibrosis grading was decreased in ERK1/2 deficient *Jak2*V617F mice vs. *Jak2*V617F settings with intact ERK1/2 24 weeks after secondary transplantation as assessed by a specialized hematopathologist blinded for the respective genotypes (n=3/group). BM reticulin (Gömöri) staining

was not evident in ERK1/2 deficient *Jak2*V617F mice, whereas moderate fibrosis was detected in *Jak2*V617F mice with intact ERK1/2. Original magnification 400x. (**P**) BM cellularity was not impaired by ERK1/2 deficiency in secondary transplantation settings at 16 weeks. (**Q**) Splenomegaly was significantly reduced by ERK1/2 deficiency 16 weeks after secondary transplantation. (**R**) Hematocrit, (**S**) reticulocytes, (**T**) leukocytes (WBC), (**U**) platelets and (**V**) CD45.2/CD45 chimerism reflecting *Jak2*V617F mutant allele burden in peripheral blood (PB), bone marrow (BM) and spleen showed maintained effects in ERK1/2 deficient settings 24 weeks after secondary transplantation. Results from one of two independent experiments are shown. Data is shown as mean ± SEM (A-N and P-V) or mean ± SD (O) and analyzed by two-tailed Student *t* test. *P≤0.05, **P≤0.01, ***P≤0.001, ****P≤0.0001.

Supplemental Figure 5

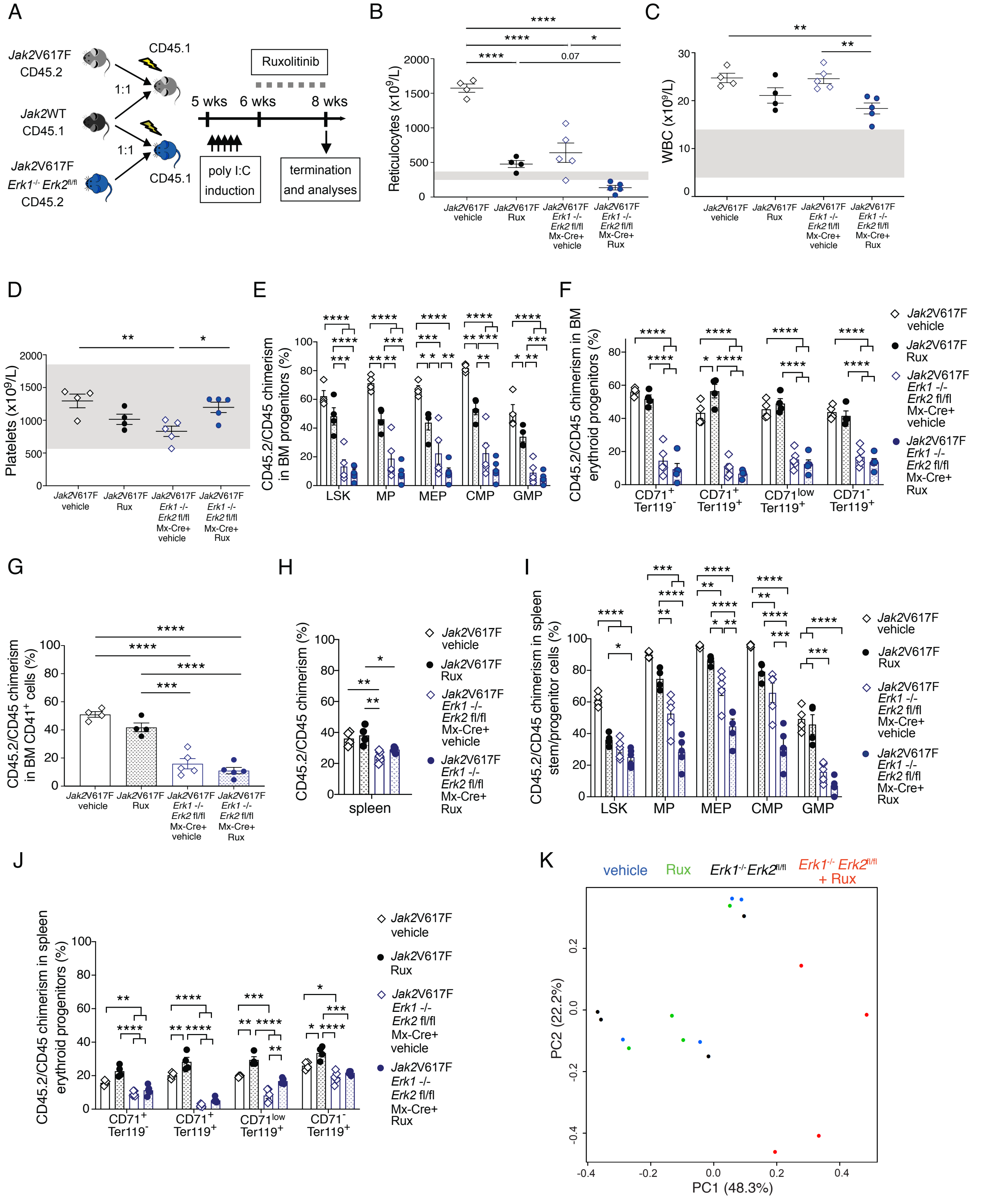

Supplemental Figure 5. Genetic targeting of ERK1/2 enhances corrective effects of JAK2 inhibition with ruxolitinib in *Jak2*V617F mice (additional data to Figure 2). (**A**) Schema of the treatment setup. *Jak2*V617F CD45.2 bone marrow (BM) with intact (grey) or deficient (blue) ERK1/2 was transplanted in 1:1 ratio with *Jak2*WT CD45.1 BM (black) into CD45.1 C57BL/6 recipient mice.

Recipient mice were treated by 5 i.p. injections of poly I:C (pIpC) 5 weeks post-transplantation followed by ruxolitinib 60 mg/kg bid for 2 weeks. (**B**) Erythrocytosis reflected by increased reticulocytes and (**C**) leukocytosis was moderated by ERK1/2 deficiency or ruxolitinib and ERK1/2 deficient settings enhanced ruxolitinib effects as indicated by further improved values upon combined treatment (n=4-5/group). (**D**) Platelets remained in the normal range upon combined ERK1/2 deficiency and ruxolitinib. (**E**) CD45.2/CD45 chimerism was also most extensively reduced by combined ERK1/2 deficiency and ruxolitinib in bone marrow (BM) myeloid stem/progenitor populations including $Lin^-Sca1^+Kit^+$ (LSK), $Lin^-Sca1^-Kit^+$ multipotent myeloid (MP), Lin^-Sca1^-c-$Kit^+FcgR^{low}CD34^-$ megakaryocyte-erythroid (MEP), Lin^-Sca1^-c-$Kit^+FcgR^{low}CD34^+$ common myeloid (CMP) and Lin^-Sca1^-c-$Kit^+FcgR^{high}CD34^+$ granulocyte-monocyte (GMP) progenitor compartments, as well as (**F**) in erythroid progenitors (n=4-5/group). (**G**) CD45.2/CD45 chimerism was also most extensively reduced by combined ERK1/2 deficiency and ruxolitinib in BM megakaryocytic cells (n=4-5/group). (**H**) CD45.2/CD45 chimerism reflecting *Jak2*V617F mutant allele burden was significantly reduced by targeting ERK1/2 in spleen (n=4-5/group). (**I-J**) CD45.2/CD45 chimerism was most extensively reduced by combined ERK1/2 deficiency and ruxolitinib in spleen myeloid stem/progenitor populations including $Lin^-Sca1^+Kit^+$ (LSK), $Lin^-Sca1^-Kit^+$ multipotent myeloid (MP), Lin^-Sca1^-c-$Kit^+FcgR^{low}CD34^-$ megakaryocyte-erythroid (MEP), Lin^-Sca1^-c-$Kit^+FcgR^{low}CD34^+$ common myeloid (CMP) and Lin^-Sca1^-c-$Kit^+FcgR^{high}CD34^+$ granulocyte-monocyte (GMP) progenitor compartments, as well as in erythroid progenitors (n=4-5/group). (**K**) Principal component analysis (PCA) of ERK1/2 target gene expression in *Jak2*V617F BM showed most extensive effects in principal component 1 (PC1) upon combined ERK1/2 deficiency and ruxolitinib (n=4/group). Data shown as mean ± SEM and analyzed by one-way ANOVA. $^*P \leq 0.05$, $^{**}P \leq 0.01$, $^{***}P \leq 0.001$, $^{****}P \leq 0.0001$.

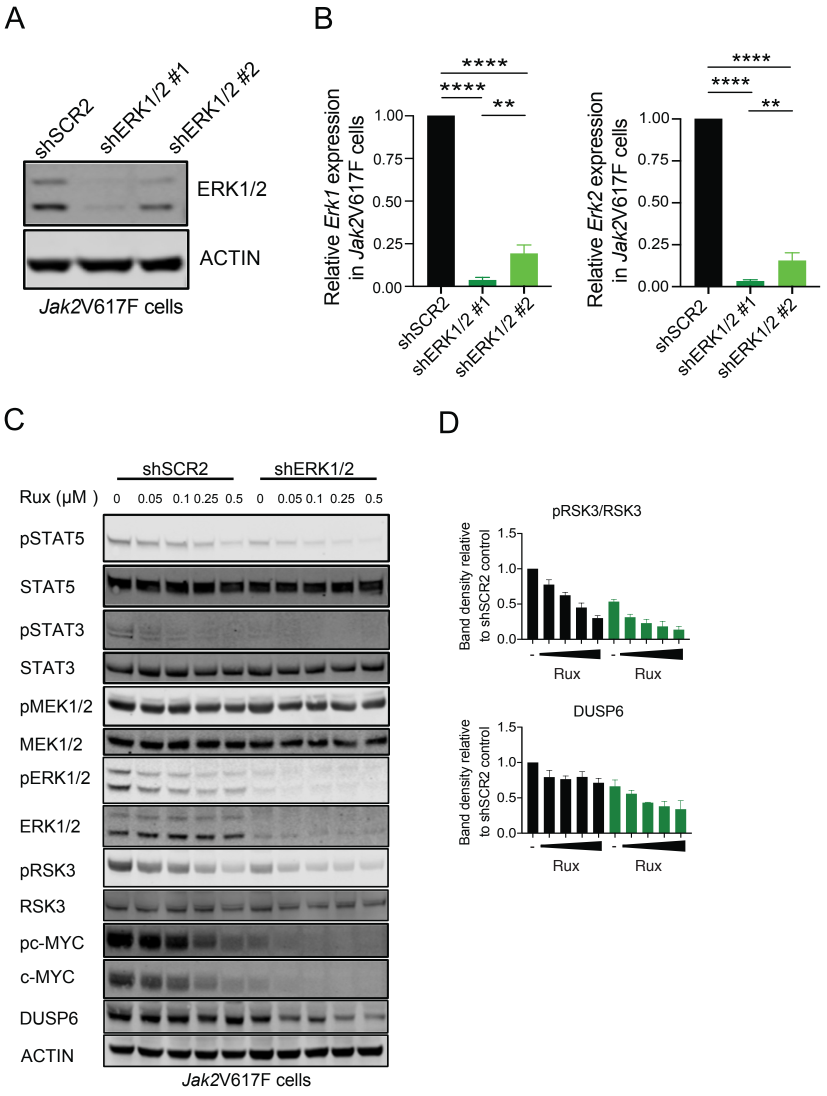

Supplemental Figure 6. ERK1/2 knockdown increases susceptibility to JAK2 inhibition in *Jak2*V617F cells (additional data to Figure 3A). (**A**) shRNA-mediated genetic targeting of ERK1/2 in *Jak2*V617F Ba/F3 cells by 2 different hairpins #1 and #2 effectively decreased ERK1/2 expression on protein and (**B**) RNA level. (**C**) Depletion of ERK1/2 affected ERK1/2 downstream targets in *Jak2*V617F Ba/F3 cells with reduced activation of RSK3 reflected by pRSK3, and decreased levels of DUSP6 and c-MYC in the absence of ruxolitinib, and further enhanced the dose-dependent ruxolitinib effects (n=2). (**D**) Quantification of pRSK3/RSK3 and DUSP6 band intensities by densitometry indicated relative to protein levels in *Jak2*V617F cells with intact ERK1/2 (black bars) in the absence of ruxolitinib (DMSO control). Data shown as mean ± SD and analyzed by one-way ANOVA. *P ≤ 0.05, **P ≤ 0.01, ***P ≤ 0.001, ****P ≤ 0.0001.

Supplemental Figure 7

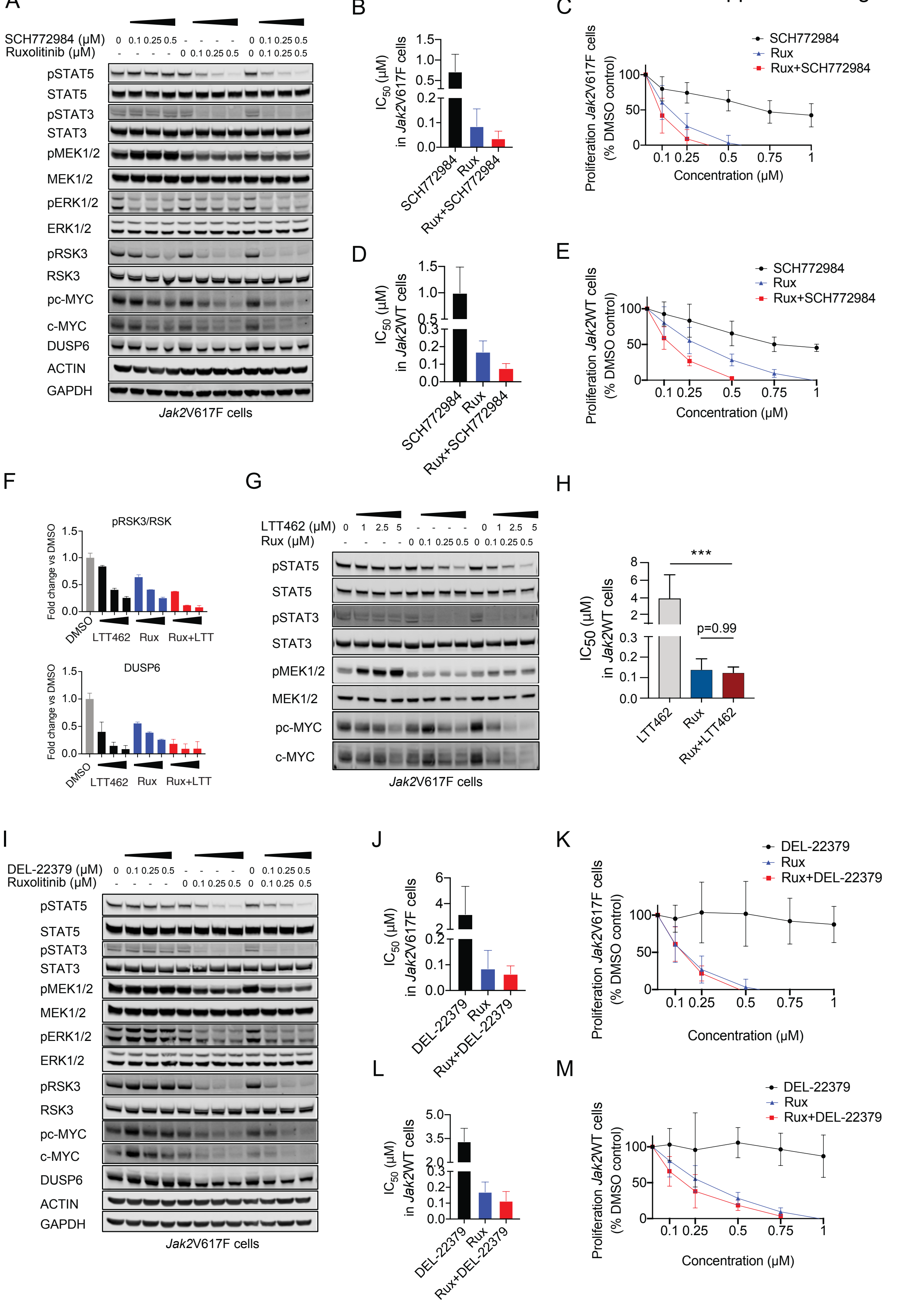

Supplemental Figure 7. ERK1/2 inhibition increases susceptibility to JAK2 inhibition in *Jak2*V617F cells (additional data to Figure 3B). (**A**) Signaling analysis by Western blot in *Jak2*V617F cells exposed to the ERK1/2 inhibitor SCH772984, the JAK2 inhibitor ruxolitinib or combined SCH772984/ruxolitinib showed enhanced inhibition of the ERK1/2 downstream targets phosphorylated RSK3 (pRSK3) and c-MYC as well as of phosphorylated ERK1/2 (pERK1/2) itself upon combined inhibitor exposure. (**B-C**) Half-maximal inhibitory concentration (IC_{50}) of ruxolitinib was decreased upon combination with the ERK1/2 inhibitor SCH772984 in *Jak2*V617F cells as illustrated by the dose-response curve (n=3). (**D-E**) Additional benefit of dual JAK2 / ERK1/2 inhibition by ruxolitinib/SCH772984 was not evident in *Jak2*WT cells with similar half-maximal inhibitory concentration (IC_{50}) upon ruxolitinib or combined ruxolitinib/SCH772984 (n=3). (**F**) Quantification of pRSK3/RSK3 and DUSP6 Western blot band intensities by densitometry is indicated relative to protein levels in *Jak2*V617F cells in the absence of inhibitors (DMSO control). (**G**) Additional signaling proteins including phosphorylated and total STAT5, STAT3, MEK1/2 and c-Myc upon exposure to the ERK1/2 inhibitor LTT462, ruxolitinib or the combination in *Jak2*V617F Ba/F3 cells (n=2). (**H**) Additional benefit of dual JAK2 / ERK1/2 inhibition by ruxolitinib/LTT462 was not evident in *Jak2*WT cells with similar half-maximal inhibitory concentration (IC_{50}) upon ruxolitinib or combined ruxolitinib/LTT462 (n=3). (**I**) Signaling analysis by Western blot in *Jak2*V617F cells exposed to the ERK inhibitor DEL-22379, ruxolitinib or combined DEL-22379/ruxolitinib did not show enhanced inhibition of ERK1/2 downstream targets upon combined inhibitor exposure (n=2). (**J-M**) Half-maximal inhibitory concentration (IC_{50}) of ruxolitinib was not clearly decreased upon combination with the ERK1/2 inhibitor DEL-22379 in *Jak2*V617F and *Jak2*WT cells as illustrated by the dose-response curves (n=2). Data shown as mean ± SD and analyzed by one-way ANOVA. $^*P \leq 0.05$, $^{**}P \leq 0.01$, $^{***}P \leq 0.001$, $^{****}P \leq 0.0001$.

Supplemental Figure 8

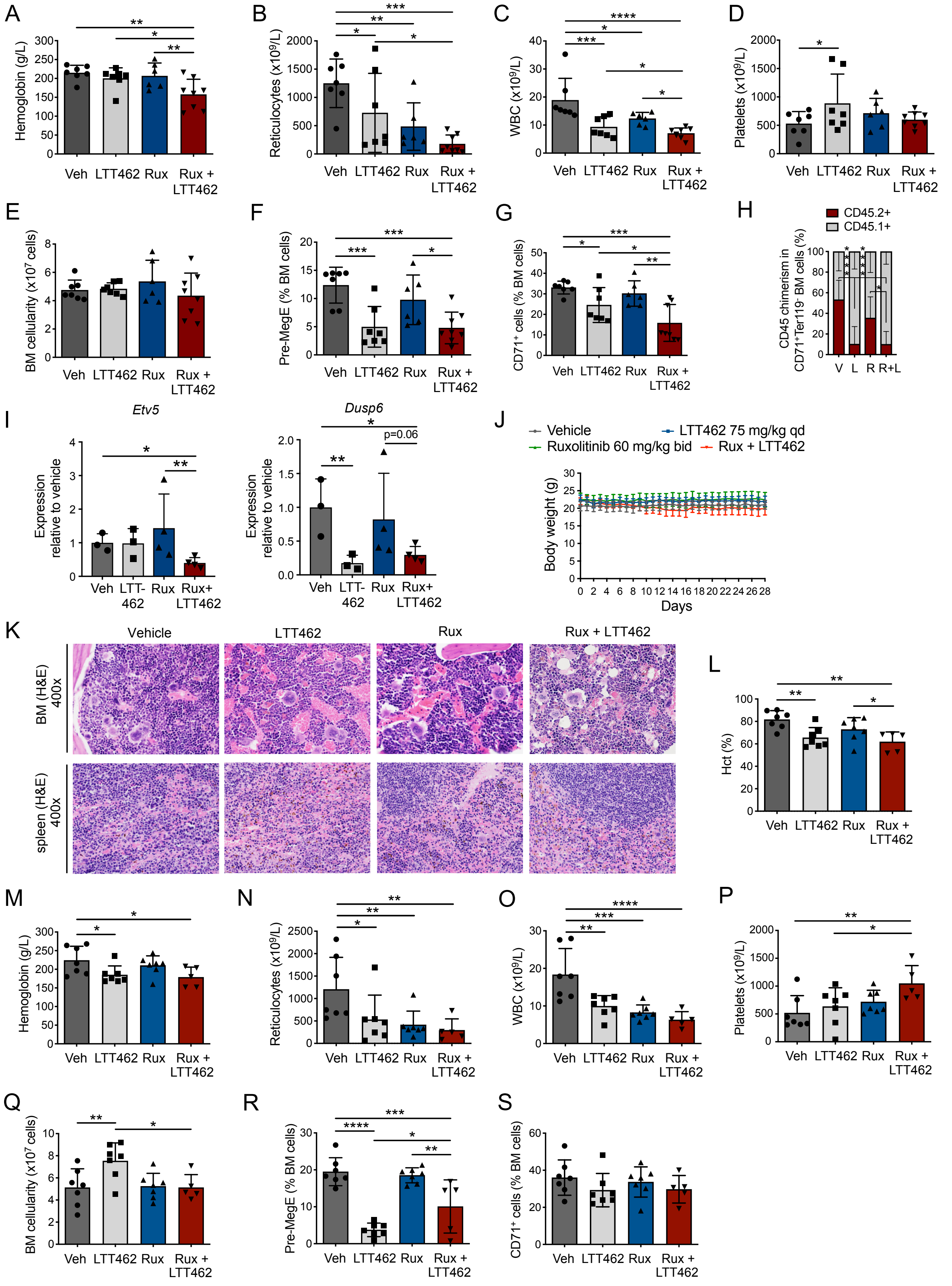

Supplemental Figure 8. Dual JAK2 and ERK1/2 inhibition by ruxolitinib/LTT462 enhances therapeutic efficacy in a *Jak2*V617F MPN preclinical model (additional data to Figure 4). (**A**) Erythrocytosis reflected by increased hemoglobin concentration and (**B**) reticulocytes was effectively corrected by combined JAK2 / ERK1/2 inhibition by ruxolitinib at 60 mg/kg bid and LTT462 at 75 mg/kg qd at 2 weeks of treatment. (**C**) White blood cells (WBC) and (**D**) platelets remained in the normal range upon treatment with LTT462, ruxolitinib or the combination after 2 weeks of treatment (n=6-8/group). (**E**) BM cellularity was not significantly lower with combined ruxolitinib/LTT462 than with either single agent therapy despite some heterogeneity within groups after 2 weeks of treatment. (**F**) Dual ruxolitinib/LTT462 enhanced reduction of BM erythroid progenitors as indicated by the $Lin^-Sca1^-c\text{-}Kit^+CD41^-FcgR^-CD150^+CD105^-$ megakaryocytic-erythroid (Pre-MegE) and (**G**) $CD71^+$ erythroid progenitor compartments as compared to ruxolitinib as a single agent after 2 weeks of treatment. (**H**) Dual ruxolitinib/LTT462 significantly improved control of the *Jak2*V617F clone reflected by CD45.2/CD45 chimerism in BM erythroid progenitors as compared to ruxolitinib monotherapy after 2 weeks of treatment. (**I**) Combined JAK2 / ERK1/2 inhibition by ruxolitinib/LTT462 effectively inhibited expression of ERK1/2 downstream target genes *Etv5* and *Dusp6* in bone marrow (BM) at 2 weeks of treatment (n=3-4/group). (**J**) Body weight was maintained upon 2 and 4 weeks of combined JAK2 / ERK1/2 inhibitor treatment. (**K**) Improved BM and spleen tissue architecture was maintained upon 2 weeks of combined JAK2 / ERK1/2 inhibitor treatment. (**L**) Erythrocytosis reflected by increased hematocrit, (**M**) hemoglobin concentration and (**N**) reticulocytes was effectively corrected by combined JAK2 / ERK1/2 inhibition by ruxolitinib at 60 mg/kg bid and LTT462 at 75 mg/kg qd at 4 weeks of treatment. (**O-P**) WBC and platelets remained in the normal range upon treatment with LTT462, ruxolitinib or the combination over 4 weeks (n=5-7/group). (**Q**) BM cellularity was not significantly lower with combined ruxolitinib/LTT462 than with ruxolitinib as a single agent after 4 weeks of treatment. (**R-S**) Dual ruxolitinib / LTT462 enhanced reduction of BM erythroid progenitors as seen for $Lin^-Sca1^-c\text{-}Kit^+CD41^-FcgR^-CD150^+CD105^-$ megakaryocytic-erythroid (Pre-MegE) and more subtly for $CD71^+$ erythroid progenitors. Data shown as mean ± SD and analyzed by one-way ANOVA. *$P \leq 0.05$, **$P \leq 0.01$, ***$P \leq 0.001$, ****$P \leq 0.0001$.

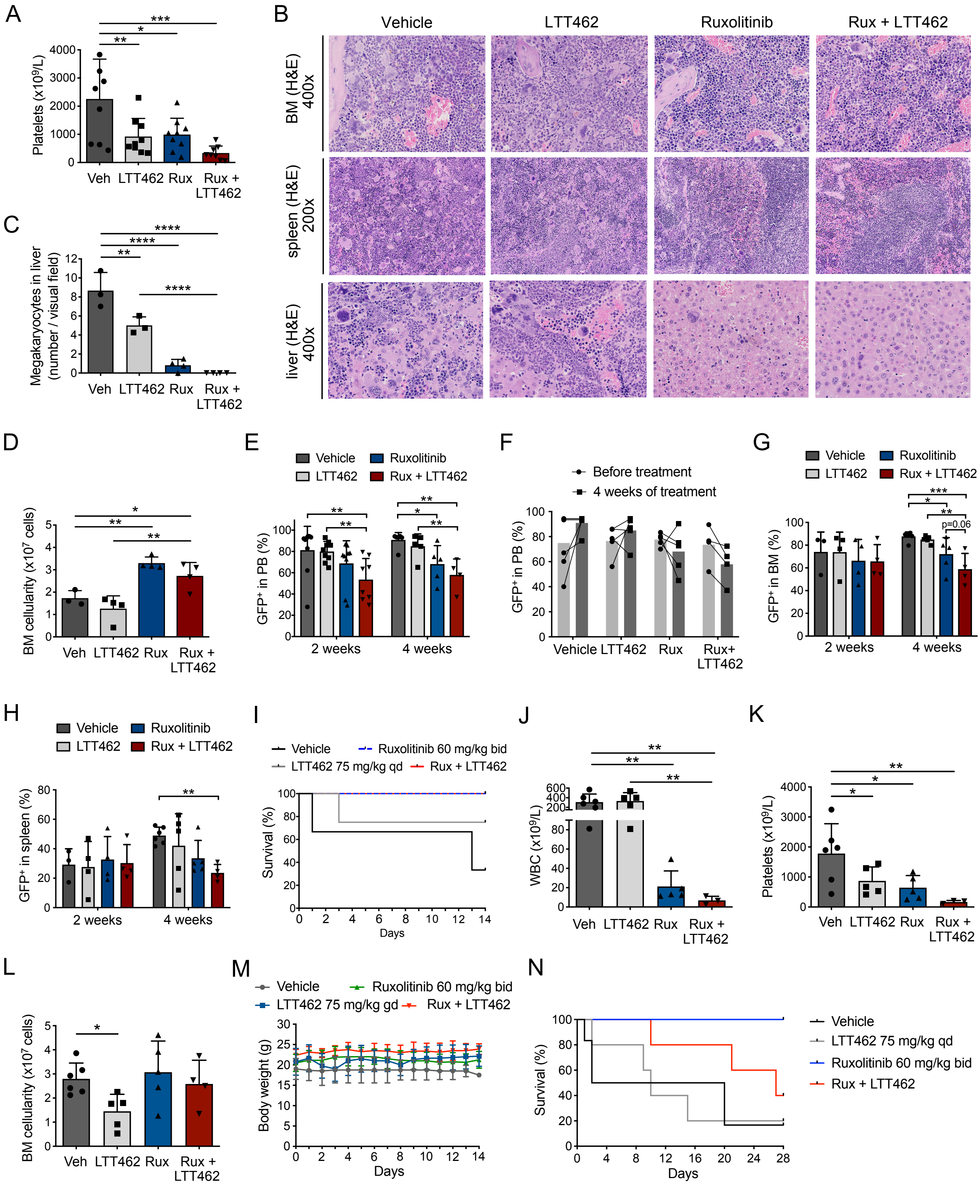

Supplemental Figure 9. Dual JAK2 and ERK1/2 inhibition by ruxolitinib/LTT462 enhances therapeutic efficacy in a *MPL*W515L MPN preclinical model (additional data to Figure 5). (A) Dual JAK2 / ERK1/2 inhibition by ruxolitinib at 60 mg/kg bid and LTT462 at 75 mg/kg qd improved platelets counts in *MPL*W515L mice after 2 weeks of treatment (n=8-9/group). (**B-C**) Combined

ruxolitinib/LTT462 improved bone marrow (BM) and spleen architecture and enhanced resolution of extramedullary hematopoiesis in the liver at 4 weeks of treatment as shown by H&E staining. Original magnification 400x for BM and liver, 200x for spleen. (**D**) BM cellularity was preserved upon ruxolitinib/LTT462 treatment in *MPL*W515L mice at 2 weeks of treatment. (**E-H**) Combined ruxolitinib/LTT462 progressively enhanced the effects of ruxolitinib as a single agent on *MPL*W515L mutant allele burden in peripheral blood (PB), BM and spleen as reflected by the percentage of GFP$^+$ cells (n=3-6/group). (**I**) Combined ruxolitinib/LTT462 improved survival of *MPL*W515L mice after 2 weeks of treatment. (**J**) Correction of leukocytosis and (**K**) thrombocytosis as well as (**L**) BM cellularity was maintained at 4 weeks of combined treatment with ruxolitinib 60 mg/kg bid and LTT462 75 mg/kg qd in *MPL*W515L mice (n=3-6/group). (**M**) Combined ruxolitinib / LTT462 was tolerable in *MPL*W515L mice. (**N**) Survival benefit was limited by occurrence of thrombocytopenia (n=6/group). Data shown as mean ± SD and analyzed by one-way ANOVA. *P ≤ 0.05, **P ≤ 0.01, ***P ≤ 0.001, ****P≤0.0001.

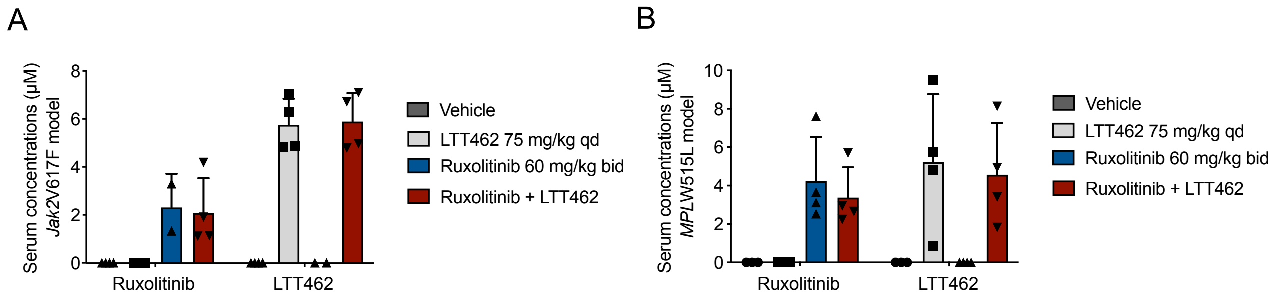

Supplemental Figure 10. Determination of serum inhibitor concentrations does not show accumulation with dual JAK2 and ERK1/2 inhibition. Serum concentrations of ruxolitinib and LTT462 were determined for the 4 treatment groups (Vehicle, LTT462 75 mg/kg qd, Ruxolitinib 60 mg/kg bid, Ruxolitinib + LTT462) at 1 week of treatment 2h after the last oral dose. (**A**) In the *Jak2*V617F mouse model, serum concentrations of ruxolitinib and LTT462 were comparable in mice treated with single agents or combined ruxolitinib / LTT462 (n=2-4/group). (**B**) In the *MPL*W515L mouse model, serum concentrations of ruxolitinib and LTT462 were comparable in mice treated with single agents or combined ruxolitinib / LTT462 (n=4/group).

Supplemental Figure 11

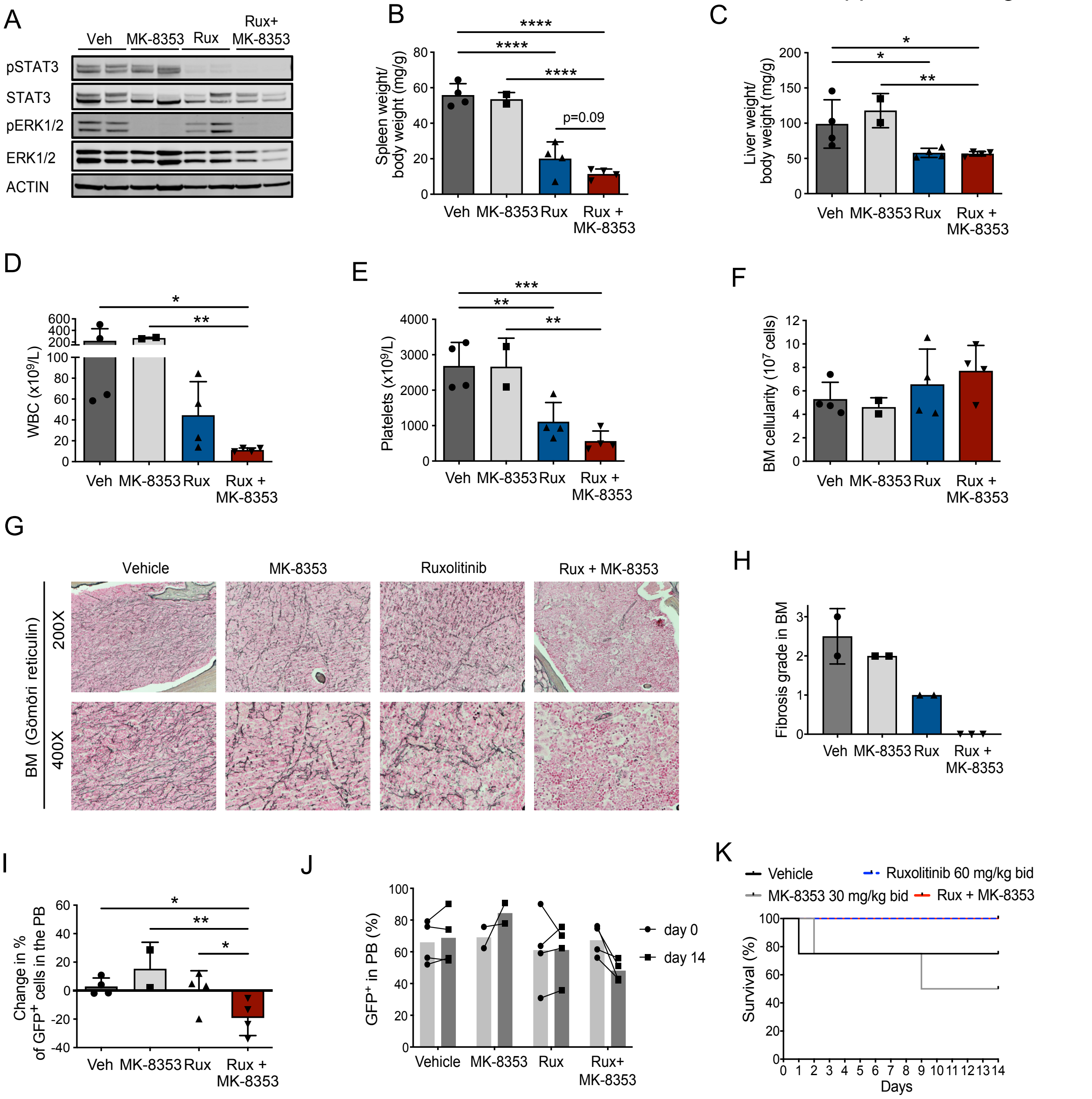

Supplemental Figure 11. Enhanced efficacy of dual JAK2 / ERK1/2 inhibition in *MPL*W515L mice is confirmed with ruxolitinib / MK-8353. (**A**) Signaling analysis in hematopoietic cells from *MPL*W515L mice treated with ruxolitinib at 60 mg/kg bid and/or MK-8353 at 30 mg/kg bid for 2 weeks showed thorough inhibition of ERK1/2 phosphorylation by MK-8353. (**B**) Dual ruxolitinib / MK-8353 enhanced reduction of splenomegaly as compared to ruxolitinib as a single agent, while (**C**) hepatomegaly was corrected by ruxolitinib. (**D**) Ruxolitinib partially corrected leukocytosis, while dual ruxolitinib / MK-8353 normalized white blood cells (WBC). (**E**) Combined ruxolitinib / MK-8353 was most effective in normalizing thrombocytosis. (**F**) Bone marrow (BM) cellularity was maintained in all treatment groups. (**G-H**) Combined ruxolitinib / MK-8353 consistently corrected BM fibrosis enhancing ruxolitinib effects as shown by reticulin (Gömöri) stain and quantified by a specialized hematopathologist blinded to treatment group assignment. Original magnification 200x-400x. (**I-K**)

Combined ruxolitinib / MK-8353 subtly and consistently reduced mutant allele burden as reflected by GFP^+ cells and improved survival of *MPL*W515L mice (n=4-5/group). Data shown as mean ± SD and analyzed by one-way ANOVA. *$P \leq 0.05$, **$P \leq 0.01$, ***$P \leq 0.00$, ****$P \leq 0.0001$.

Supplemental Figure 12

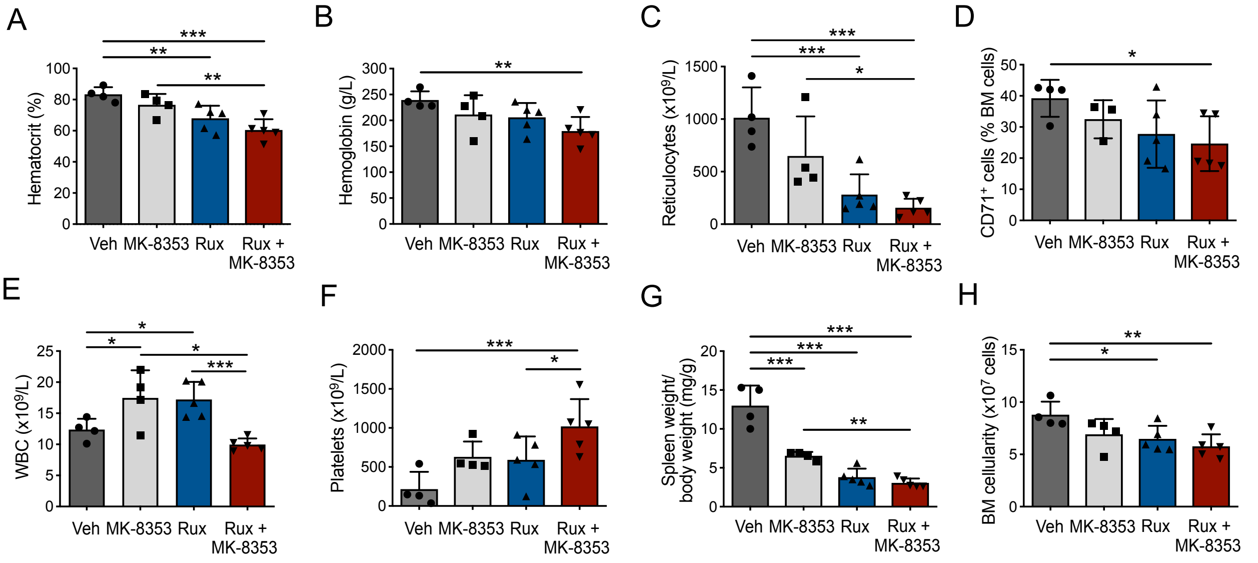

Supplemental Figure 12. Enhanced efficacy of dual JAK2 / ERK1/2 inhibition in *Jak2*V617F mice is confirmed with ruxolitinib / MK-8353. (**A**) Erythrocytosis reflected by increased hematocrit, (**B**) hemoglobin concentration and (**C**) reticulocytes was effectively corrected by combined JAK2 / ERK1/2 inhibition with 60 mg/kg ruxolitinib bid and 40 mg/kg MK-8353 bid at 2 weeks of treatment (n=4-5/group). (**D**) Combined JAK2 / ERK1/2 inhibition by ruxolitinib/MK-8353 reduced $CD71^{+}$ erythroid progenitors in bone marrow (BM). (**E-F**) White blood cells (WBC) and platelets remained in the normal range upon treatment with MK-8353, ruxolitinib or combined ruxolitinib/MK-8353. (**G-H**) Splenomegaly was corrected and BM cellularity was not significantly reduced by combined ruxolitinib/MK-8353 as compared to either single agent therapy. Data shown as mean ± SD and analyzed by one-way ANOVA. *P ≤ 0.05, **P ≤ 0.01, ***P ≤ 0.001.

Supplemental Figure 13

A

Pts	Diagnosis	Driver	VAF	Other mutations	Sex	Age	Sample	Therapy
1	PMF	*JAK2* V617F	41%	None	m	58	at diagnosis	untreated
2	PMF	*JAK2* V617F	25%	None	f	27	at diagnosis	untreated
3	PV	*JAK2* V617F	43%	None	m	71	at diagnosis	phlebotomy
4	PV	*JAK2* V617F	8%	None	m	39	at diagnosis	phlebotomy
5	ET	*JAK2* V617F	4%	None	m	75	at diagnosis	untreated
6	ET	*JAK2* V617F	12%	None	f	29	at diagnosis	untreated

B

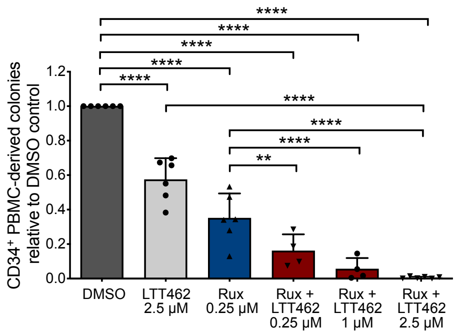

C

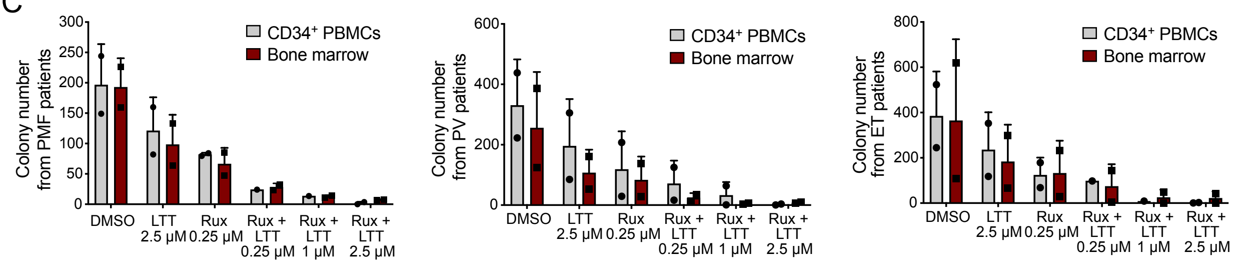

D

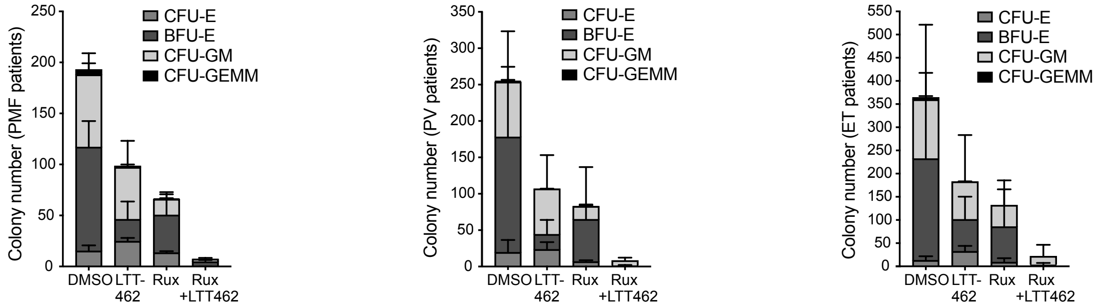

E

Pts	Diagnosis	Driver	VAF	Other mutations	Sex	Age	Sample	Therapy
1	ET	*MPL* W515L	23%	None	m	74	at diagnosis	untreated
2	PMF	*MPL* W515K	18%	None	m	63	at diagnosis	untreated
3	PMF	*MPL* W515K	62%	*ASXL1, CBL*	m	58	at diagnosis	untreated
4	PMF	*MPL* W515R	28%	None	f	66	at diagnosis	untreated

F

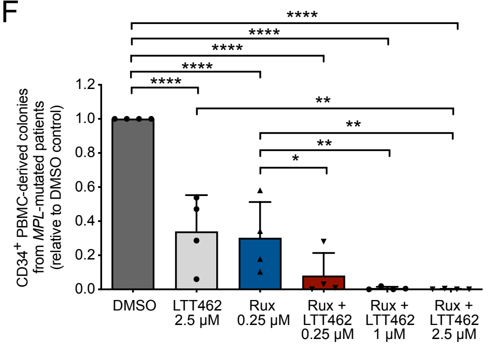

G

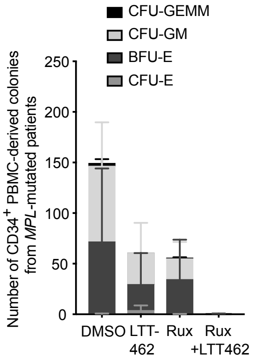

H

Healthy donor	Sex	Age
1	m	34
2	m	35
3	m	38
4	m	32

I

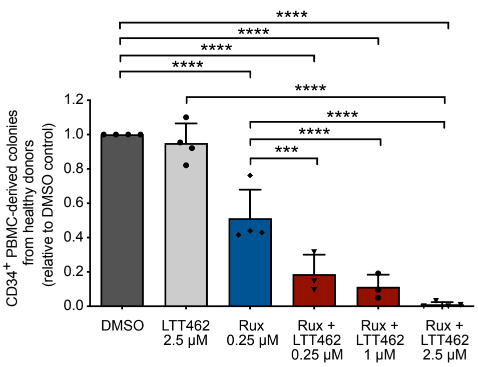

J

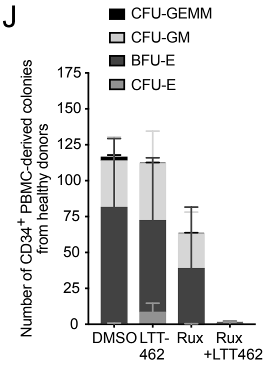

Supplemental Figure 13. Dual JAK2 and ERK1/2 inhibition enhances suppression of myeloid colony formation and ERK1/2 target activation from primary *JAK2* and *MPL* mutated patient cells (additional data to Figure 6). (**A**) Paired blood and bone marrow (BM) isolates from *JAK2*V617F mutated MPN patients including primary myelofibrosis (PMF, n=2), polycythemia vera (PV, n=2) and essential thrombocythemia (ET, n=2), which were obtained at primary diagnosis in the absence of cytoreductive therapy, were assessed for the potential of myeloid colony outgrowth upon exposure to dual JAK2 and ERK1/2 inhibition by ruxolitinib/LTT462. (**B**) Peripheral blood mononuclear cells (PBMCs) from *JAK2*V617F mutated MPN patients were enriched for $CD34^+$ cells, seeded into methocult at 3000 cells/well and scored after 10 days. ERK1/2 inhibition by LTT462 at 0.25, 1 and 2.5 µM improved control of myeloid colony formation from *JAK2*V617F mutated $CD34^+$ cells seen with ruxolitinib at 0.25 µM in a dose-dependent manner (n=6). (**C**) Combined ruxolitinib/LTT462 was most effective in preventing myeloid colony outgrowth in the three MPN subtypes including PMF, PV and ET (n=2/entity). (**D**) Improved corrective effects by dual ruxolitinib/LTT462 were present in all the assessed colony subtypes derived from BM cells including erythroid (CFU-E, BFU-E), granulocyte-macrophage (CFU-GM) and granulocyte-erythroid-macrophage-megakaryocyte (CFU-GEMM) in PMF, PV and ET subsets (n=2/entity). (**E**) Peripheral blood mononuclear cells (PBMCs) from *MPL* mutated MPN patients, which were obtained at primary diagnosis in the absence of cytoreductive therapy, were enriched for $CD34^+$ cells, seeded into methocult at 3000 cells/well and scored after 10 days. (**F**) ERK1/2 inhibition by LTT462 at 0.25, 1 and 2.5 µM improved control of myeloid colony formation from *MPL* mutated $CD34^+$ cells seen with ruxolitinib at 0.25 µM in a dose-dependent manner (n=4). (**G**) Improved corrective effects by dual ruxolitinib/LTT462 were present in all the assessed colony subtypes derived from *MPL* mutated $CD34^+$ cells, including erythroid (CFU-E, BFU-E), granulocyte-macrophage (CFU-GM) and granulocyte-erythroid-macrophage-megakaryocyte (CFU-GEMM) (n=4). (**H**) Peripheral blood from healthy donors was collected to assess the potential of myeloid colony outgrowth upon exposure to dual JAK2 and ERK1/2 inhibition by ruxolitinib/LTT462 (n=4). (**I**) Peripheral blood mononuclear cells (PBMCs) from healthy donors were enriched for $CD34^+$ cells, seeded into methocult at 3000 cells/well and scored after 10 days. ERK1/2 inhibition by LTT462 at 0.25, 1 and 2.5 µM reduced the growth of myeloid colonies from healthy $CD34^+$ cells seen with ruxolitinib at 0.25 µM in a dose-dependent manner (n=4). (**J**) Inhibitory effects by dual ruxolitinib/LTT462 were present in all the assessed colony subtypes derived from healthy $CD34^+$ cells, including erythroid (CFU-E, BFU-E), granulocyte-macrophage (CFU-GM) and granulocyte-erythroid-macrophage-megakaryocyte (CFU-GEMM) (n=4). Data are presented as mean ± SD. $^*P \leq 0.05$, $^{**}P \leq 0.01$, $^{***}P \leq 0.001$, $^{****}P \leq 0.0001$ by one-way ANOVA.
